# One-Degree-of-Freedom Mechanical Metamaterials with Arbitrary Prescribability and Rapid Reprogrammability of Force–Displacement Curves

**DOI:** 10.34133/research.0715

**Published:** 2025-06-09

**Authors:** Hui Li, Wei Li, Huixin Yang, Joseph M. Gattas, Qingyang Chen, Yang Li

**Affiliations:** ^1^ The Institute of Technological Sciences, Wuhan University, Wuhan, Hubei 430072, China.; ^2^ Hongyi Honor College, Wuhan University, Wuhan, Hubei 430072, China.; ^3^ School of Power and Mechanical Engineering, Wuhan University, Wuhan, Hubei 430072, China.; ^4^ School of Civil Engineering, The University of Queensland, Brisbane 4072, Australia.; ^5^ Wuhan University Shenzhen Research Institute, Wuhan, China.

## Abstract

Mechanical metamaterials, by introducing porous structures into the materials, can achieve complex nonlinear responses through the large deformation of structures, which support a new generation of impact energy absorption and vibration damping systems, wearable electronics, and tactile simulation devices. However, arbitrarily customizable stress–strain curves have yet to be achieved by existing mechanical metamaterials, which are inherently multi-degree-of-freedom (multi-DOF) deformable systems, and their deformation sequence is influenced by the minimum energy gradient principle. Multi-DOF metamaterials behave like underactuated systems, where the number of degrees of freedom exceeds the number of actuators. As a result, their deformation is controlled by the material’s elastic forces, inertial forces, and boundary constraints. Here, we propose a novel composition of elastic components integrated with one-degree-of-freedom (1-DOF) kinematic bases, forming a fully actuated system in which the number of actuators equals the number of degrees of freedom. The deformation of each elastic component is governed by its designed 1-DOF kinematic path. Consequently, the stress–strain profile can be arbitrarily prescribed, for instance, controlled multistage strain softening curve is achievable, as the principle of minimum energy gradient does not affect the deformation sequence dictated by the 1-DOF kinematic base. Furthermore, a class of shape memory alloys (SMAs) is introduced as active components to enable rapid in situ property change, providing versatility in switching between different target responses. The analytical inverse design method, numerical analysis, parametric study of different target responses, and experimental validation are carried out. Lastly, preliminary demonstrations of designable anisotropic nonlinear responses are presented.

## Introduction

The stress–strain curve is a fundamental property of materials, which contains information on stiffness, strength, elongation, strain-hardening or softening effect, etc. Mechanical metamaterials introduce porous structures that bend and buckle when deformed and can generate a highly designable mechanical response [1–4], which enhances designability due to the rich topological [5–8] and geometric parameters of the introduced mechanical structures. This customized nonlinear mechanical response can be useful in vascular scaffolds to promote tissue regeneration with a comparable nonlinear mechanical property of surrounding natural tissue [9]. Another type of application is in flexible sensors [10] and biomedical devices [11] in wearable electrics [12–15]. Such design mechanical response can also be used in energy-absorbing components in vehicles with dramatic strain-hardening effect that are soft in short-stroke contact with pedestrians, but can be very strong in large-stroke collision to protect occupants [16,17], and multi-stage zero-stiffness vibration isolators [18–23] mitigating ultra-low frequency vibration. The forms of the porous structures introduced are foams, cellular units, graded and hierarchical structures, bistable unit-cell assembly [24], microlattice [25,26], minimum curvature surfaces [27], Miura-ori stacking [28], kinematic linkages [29,30], etc. Such porous structures have multiple potential deformation modes, and the mode corresponding to the minimum energy gradient shall occur. This results in a strain-hardening effect in a graded structure, as the softer layers always deform prior to the stiffer layers, regardless of how they are arranged. Some unit cells have buckling behavior that can result in local strain softening [31] or one-time softening [32]; overall stress–strain curves still have strain-hardening profiles [33,34]. Detailed illustration of this limitation is provided in this paper. There is one exception in which slender unit cells with parallel coupling are used [18]. Since the slender unit cells are independent from each other in the loading direction, there is no room for the principle of minimum energy gradient to take effect. As a result, such parallel coupling requires mechanical sliders to provide side supports for those slender unit cells, leading to bulky devices.

There are different inverse design methods suitable for the respective conceptual constructions. For multi-degree-of-freedom (DOF) deformable systems [35–40], due to the involvement of nonlinear large deformation and the uncertainty of the deformation mechanism, it is most suitable to run a large number of forward analysis, build a database and a surrogated empirical model, and then perform the inverse design accordingly [32,41–46]. For 1-DOF deformable systems where the deformation mechanism is determined, an analytical kinematic model can be derived that eventually leads to a constrained optimization problem. Mechanical metamaterial manufacturing usually uses 3-dimensional (3D) printing and mechanical assembly on a macroscale and then seeks miniaturization.

**Fig. 1.**
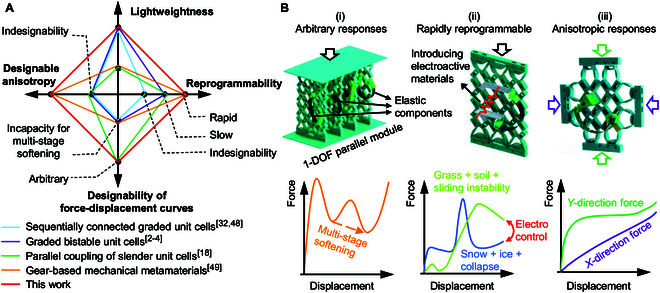
Comparison of the proposed mechanical metamaterials with previous works. (A) Comparison terms of force–displacement curve designability, reprogrammability, lightweightness, and designable anisotropy. (B) Schematic diagram illustrating the potential of our work in relation to arbitrary responses, rapidly reprogrammable responses, and anisotropic responses.

In summary, there are 4 main conceptual designs to achieve the prescribed force–displacement curves, as shown in Fig. 1A. The first category comprises sequentially connected graded unit cells [47] designed for single monotonical strain-hardening or a one-time softening response [32,48]. The second type involves graded bistable unit cells for sinusoidal response, where the valleys of the sinusoidal curves remain constant or increase with deformation (incapable of multi-stage softening) [2–4]. The third type employs parallel coupling of slender unit cells through slideways to achieve arbitrary reaction curves [18]. The fourth type uses gears with encoded stiffness gradients, which allows for rapid reprogramability and anisotropic designability [49], but it cannot accommodate arbitrary stress–strain curves. In this paper, we propose a novel conceptual design that couples elastic components with 1-DOF kinematic bases, which is named “1DOFmat”. There are other classic one-degree-of-freedom (1-DOF) unit cell [50] structures such as triangular [51], square [52], and polygonal [53,54]. Additionally, different topological connections [55,56] and 3D kinematic bases [57] are considered (see Section S1.2). Flexible hinges can alternatively be realized using textile hinges [58], offering improved resistance to tensile forces and in-plane twisting. Our approach represents the simplest and most foundational concept, introducing a metamaterial concept that couples 1-DOF kinematic bases with elastic components [59]. The deformation of each elastic component is dictated by the designed 1-DOF motion, leaving no room for internal energy-minimizing adaptation, resulting in an arbitrarily customizable nonlinear response where multi-stage strain softening can be achieved.

Moreover, this paper presents a rapidly reprogrammable mechanical metamaterial that integrates a 1-DOF kinematic base, elastic components, and electrical control. Two distinct perceptual scenarios are explored: positive stiffness in grass and soil, and negative stiffness associated with sliding instability during walking, along with multi-stage negative stiffness in snow and ice, where large negative stiffness occurs during collapse. Smart materials, such as shape memory alloys (SMAs), piezoelectric materials, magneto-sensitive materials, and liquid crystal elastomers, can sense and respond to external stimuli, typically exhibiting adaptive changes in response to environmental conditions. The method proposed in this paper is the only one that can achieve arbitrarily customizable reaction curves in a lightweight manner, as well as rapidly reprogrammable and anisotropic responses, as shown in Fig. 1B.

## Results

In this paper, the fundamental problem of multi-DOF deformable systems in prescribing arbitrary responses is illustrated. Then, the geometric construction of the new design (1DOFmat) is given, and a simple analytical model is derived, allowing for a quick inverse design with respect to different nonlinear target responses. Manufacturing processes involving 3D printing and mechanical assembly, experiments, and a parametric study of various target curves are provided. Finally, preliminary demonstrations of SMA-based rapidly reconfigurable metamaterials for zero-stiffness vibration and virtual reality (VR) tactile simulation, and designable anisotropic nonlinear responses are presented.

### Comparing the ability of 1-DOF and multi-DOF deformable systems to prescribe arbitrary responses

This section introduces the differences between 1-DOF and multi-DOF deformable systems in terms of their ability to prescribe arbitrary responses. Multi-DOF deformable metamaterials behave like underactuated systems, where the number of degrees of freedom exceeds the number of actuators. In these systems, deformation is governed by elastic forces, inertial forces, and boundary constraints, with the kinematic path in the energy landscape following the principle of minimum energy gradient. However, 1-DOF systems behave like fully actuated systems, where the number of degrees of freedom equals the number of actuators. The deformation is completely determined by the 1-DOF kinematic base, allowing for arbitrary design of the kinematic path without being constrained by the energy gradient principle. To explore this limitation, this section analyzes the energy landscape of both linear and nonlinear multi-DOF systems and 1-DOF systems under displacement loading, providing insight into their ability to achieve arbitrary responses.

**Fig. 2.**
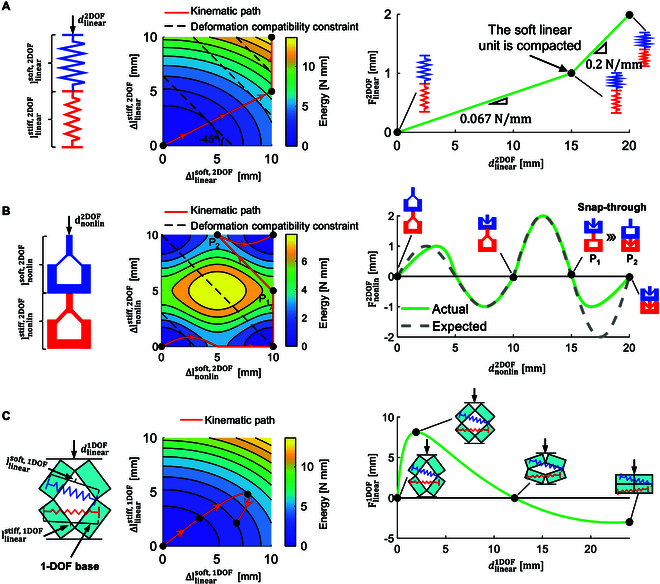
The capability of 1-DOF and multi-DOF deformable systems to achieve arbitrary responses. (A) Force–displacement curves for 2 linear springs in series with different stiffness, where the blue and red lines represent soft and stiff spring, respectively. (B) Force–displacement curves for 2 nonlinear units in series. State $P_{1}$ snaps to state $P_{2}$ by adhering to the principle of minimum energy gradient in a multi-DOF deformable system. The green solid line represents the actual reaction force curve, while the gray dashed line indicates the expected force–displacement curve. (C) Force–displacement curves for a 1-DOF deformable system, where the kinematic path can traverse the energy landscape arbitrarily.

Firstly, consider a 2-DOF deformable system consisting of 2 linear springs, as shown in Fig. 2A. The blue spring, representing the soft spring, has a stiffness of $0.1\;N\;{\mathrm{mm}}^{-1}$, while the red spring, representing the stiff spring, has a stiffness of $0.2\;N\;{\mathrm{mm}}^{-1}$. Both springs have a compression limit of 10 mm. The energy landscape during the compression process of the linear series springs is shown in the middle of Fig. 2A. The horizontal and vertical axes represent the deformations of the soft and stiff springs, respectively, and the color indicates the total elastic energy stored in the 2 springs. The black solid lines represent equal-energy contours, while the −45° black dashed line represents the deformation compatibility constraint. The solid red line with arrows indicates the kinematic path traced by changing the total displacement $d_{\text{linear}}^{2\mathrm{DOF}}$ from 0mm to 20mm. The kinematic path intersects the point of tangency between the deformation compatibility constraint and the equal-energy contours at nonboundary regions. When the loading displacement reaches 15 mm, the soft spring reaches its compression limit of 10 mm. The stiffness of the 2 series-connected springs is $0.067\;N\;{\mathrm{mm}}^{-1}$ (see the right side of Fig. 2A), which is lower than the stiffness of the soft spring ($0.1\;N\;{\mathrm{mm}}^{-1}$). After the soft spring reaches its stroke limit, only the stiff spring deforms, and the overall stiffness of the series-connected springs becomes equal to the stiffness of the stiff spring, $0.2\;N\;{\mathrm{mm}}^{-1}$ (see Section S3.1 and Movie S1). The kinematic path of the 2-DOF linear deformable system remains linear, inhibiting the generation of nonlinear responses.

Another example worth considering is the system of 2 nonlinear bistable units with different stiffnesses, connected in series, as shown in Fig. 2B. These bistable nonlinear units are assumed to follow 2 different sinusoidal functions (detailed information is provided in Section S3.2, Movie S1, and Code S1). The force–displacement curve of the series-connected nonlinear units demonstrates that the valleys of the total force–displacement curve do not decrease, as the stiff unit snaps and pulls the soft unit in an impulsive manner from state $P_{1}$ to $P_{2}$, instantly releasing elastic energy. $P_{2}$ is the minimum energy point of the deformation compatibility constraint at $d_{\text{nonlin}}^{2\mathrm{DOF}}=15\;\mathrm{mm}$. This reveals the challenge of achieving multi-stage softening in multi-DOF metamaterials, as the minimum energy gradient principle governs the deformation in multi-DOF deformable systems.

A 1-DOF deformable system, consisting of 2 linear springs coupled with 4 rotating rectangles, is introduced in Fig. 2C. The system’s kinematic path is a customizable curve that can traverse the energy landscape arbitrarily, free from deformation compatibility constraints and not bound by the principle of minimum energy gradient. As a result, 1-DOF systems exhibit a stronger ability to prescribe arbitrary responses compared to multi-DOF systems.

### Conceptual design of mechanical metamaterials based on elastic components coupled with kinematic bases of 1-DOF (1DOFmat)

**Fig. 3.**
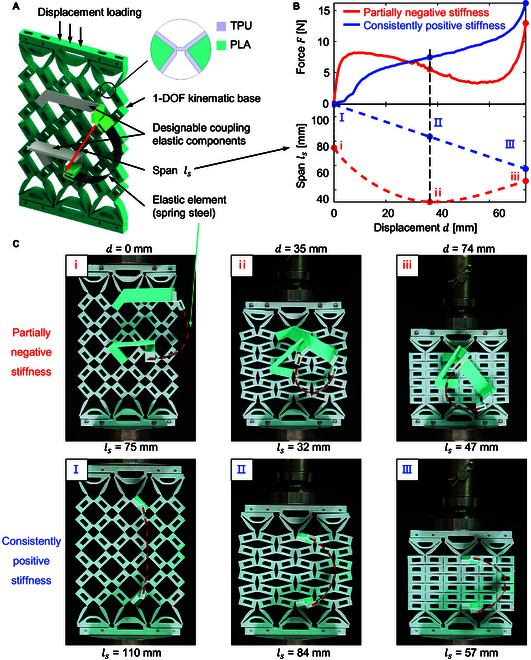
The conceptual design of 1DOFmat. (A) Physical platform of coupling elastic components with 1-DOF kinematic bases (1DOFmat), where $l_{s}$ represents the span of the elastic beam under displacement loading. (B) Graphs of the correlation of the response force *F* (obtained from experiments) and the span $l_{s}$ for 2 distinctive 1DOFmats, where one exhibits a nonmonotonic span ($l_{s}$) and corresponds to partially negative stiffness, and the other one demonstrates monotonic span ($l_{s}$) with consistently positive stiffness. (C) Experimental snapshots of the negative and positive stiffness 1DOFmats at the displacement of 0, 35, and 74 mm, respectively.

To address the problem shown above, this paper employs a 1-DOF kinematic frame to couple elastic components to eliminate the room for the energy gradient to take effect in the deformation sequence. The conceptual design named 1DOFmat is shown in Fig. 3A. The kinematic base consists of hinged quadrilateral units arranged in a 6 × 6 grid. Each unit is a rectangular hinged quadrilateral measuring 20 mm in length, 10 mm in width, and 10 mm in thickness. In addition, each small hinged quadrilateral unit has a rectangular hole of 10 mm length and 5 mm width in the center of the block (Section S1). Rectangular units are joined by flexure hinges, printed using thermoplastic polyurethane (TPU) elastic material with dimensions of 1 mm in length, 0.4 mm in thickness, and 10 mm in width. The inner part of the rectangular block is made of a rigid plastic, polylactic acid (PLA), while the outer part is covered with a layer of TPU. This dual material approach [60] is designed to achieve the desired mechanical properties, with the TPU layer providing low stiffness for the flexible hinge and the underlying PLA structure providing robust support for the connected elastic components.

The 6 × 6 hinged quadrilaterals have an inverted trapezoidal block with upper and lower connections sliding left and right relative to the upper and lower strip plates, both of which are fitted with 4 bearings to ensure friction-free energy changes during compression of the 1-DOF kinematic base (Section S2). The elastic component consists of spring steel and 2 elastomeric couplers, which are fixed to the through holes of the rectangular block of the kinematic base. The elastomeric coupler is designed with a pin securely attached at one end to the through-hole of the rectangular unit in 1-DOF kinematic bases. At the other end, a TPU flex hinge is firmly connected to the spring steel. Under displacement loading, the 1-DOF rectangular unit undergoes rotation, leading to a variation in the span ($l_{s}$) between the 2 end points of the spring steel.

Each elastic component is defined by 2 integer variables and 6 real variables; displacement loading of the metamaterial induces rotation in the 1-DOF kinematic base, altering the span $l_{s}$ between the spring steel end points. Consequently, the spring steel bends more or less, causing the energy and reaction force to change. Adjusting the design variables of each elastic component allows precise control of steel deformation and bending, thereby controlling the reaction force. The inverse design of reaction forces is detailed in the next section and in Section S4.

We present 2 different 1DOFmats with distinct force–displacement curves (see Fig. 3B and Movie S2). The blue line represents a positive stiffness curve (monotonic increasing curve), while the red line illustrates a partially negative stiffness curve (nonmonotonic curve). The blue and red dashed lines show how the span ($l_{s}$) between the 2 end points of the spring steel changes under displacement loading for positive and partially negative stiffness 1DOFmats, respectively. The positive stiffness of the 1DOFmat spring steel exhibits a linear decrease in the distance between its 2 end points, indicating stored energy. In contrast, the partially negative stiffness 1DOFmat spring steel initially undergoes an increase in distance followed by a decrease, suggesting both energy storage and subsequent release. The spring steel for positive and partially negative stiffness 1DOFmats shares characteristics such as Young’s modulus *E* = 205 GPa, initial length $L_{0}$ = 115 mm, width *w* = 10 mm, and thickness *b* = 0.3 mm. The deformation diagrams corresponding to loading displacements of *d* = 0 mm, *d* = 35 mm, and *d* = 74 mm are presented in Fig. 3C. The metamaterial transforms energy primarily through the rotation of the flexible hinge in the 1-DOF kinematic base and the bending deformation of the spring steel in the elastic assembly. By determining the response of the 1-DOF kinematic base to displacement loading and adjusting the spring steel specifications, we can control the trend of change in span ($l_{s}$) between the 2 end points of the spring steel. This control allows for the precise regulation of elastic energy changes during loading displacement, which affects the force–displacement curve. Ultimately, this adjustment enables the creation of metamaterials with customized stress–strain curves, offering versatility in designing materials with desired mechanical characteristics.

### Inverse design method

Each kinematic base contains 36 holes. The relative coordinates of the first elastic component support in terms of the local coordinates of the rectangular block at index $N_{1}$ are denoted as $u_{1}$ and $v_{1}$, while $u_{2}$ and $v_{2}$ refer to the second elastic component support at index $N_{2}$ (Sections S4 and S5). The rest of the spring steel on the elastic component is $L_{0}$, with w representing its width. Considering the rigidity of the 3D-printed kinematic base used in our 1DOFmat, we selected spring steel with a thickness of 0.3 mm to achieve an optimal balance between flexibility and rigidity. Each rectangular block undergoes rotational movement, which causes the attached elastic components to bend accordingly.

The fundamental process of inverse design involves the following steps:

Preliminary selection: The required number of elastic components $N_{\text{elas}}$ and 1-DOF kinematic bases $N_{\text{base}}$ are pre-selected based on the complexity of the target curve. A specific number of sample points $N_{\text{test}}$ is selected from the target curve for fitting.

Parameter expression: The parametric representation of the elastic components expresses the energy of the elastic potential in accordance with the established kinematic relationships. The elastic energy stored in the components is a function of their geometric properties and the coupling with the rectangular through-holes. The reaction force $F_{1\text{DOFmat}}$ is determined as the sum of the base kinematic force $F_{\text{base}}$ (derived from compression experiment) and the elastic force $F_{\text{elas}}$, derived from the derivative of the elastic potential energy $E_{\text{elas}}^{\text{total}}$ of the elastic components with respect to displacement loading $d_{\text{disp}}$:

$$\begin{array}{c} E_{\text{elas}}^{\text{total}}=\sum_{i=1}^{N_{\text{elas}}}E_{\text{elas}}^{i}\left(N_{1}^{i},\;N_{2}^{i},\;u_{1}^{i},\;v_{1}^{i},\;u_{2}^{i},\;v_{2}^{i},\;L_{0}^{i},\;w^{i},\;d_{\text{disp}}\right), \end{array}$$ (1)

$$F_{1\text{DOFmat}}=F_{\text{base}}+\frac{dE_{\text{elas}}^{\text{total}}}{dd_{\text{disp}}}.$$ (2)

Minimization: The objective is to minimize the squared sum of the differences between the reaction force fitted at the sampling points and the target reaction force. Inequality constraints are imposed on the span between the 2 end points of the spring steel, ensuring that its bending remains within the elastic range. The design problem for the elastic components can be formulated as a constrained optimization problem:

$$\begin{array}{c} \begin{array}{ll} \text{for}:\; & X_{p}^{i}=\left[N_{1}^{i},\;N_{2}^{i},\;u_{1}^{i},\;v_{1}^{i},\;u_{2}^{i},\;v_{2}^{i},\;L_{0}^{i},\;w^{i}\right],\\ \text{Minimize}:\; & L=\sum_{j=1}^{N_{\text{test}}}{\left(F_{1\text{DOFmat}}^{j}-F_{\text{target}}^{j}\right)}^{2},\\ \text{Subject to}:\; & L_{\text{plastic}}^{i}<l_{s}<L_{0}^{i},\\ & 1\leq N_{1}^{i},N_{2}^{i}\leq 36,\\ & \mathrm{lb}\leq u_{1}^{i},v_{1}^{i},u_{2}^{i},v_{2}^{i},L_{0}^{i},w^{i}\leq \mathrm{ub}, \end{array} \end{array}$$ (3)

where $N_{\text{test}}$ is the number of sample points, $F_{\text{target}}^{j}$ is the target response curve, and $L_{\text{plastic}}^{i}$ is the distance of plastic deformation in the spring steel of elastic components. lb and ub represent the lower and upper bounds of the variables. The optimization problem can be solved using the fmincon function in MATLAB.

Accuracy assessment: The mean relative error (MRE) serves as the evaluation metric for the accuracy of the fitting of the customized curve:

$$\text{error}=\frac{1}{N_{\text{test}}}\sum_{j=1}^{N_{\text{test}}}\left|\frac{F_{\text{target}}^{j}-F_{1\text{DOFmat}}^{j}}{F_{\text{target}}}\right|.$$ (4)

If the MRE is below the specified error ${\text{error}}_{\text{target}}$, the design parameters of the elastic components are finalized. However, if it exceeds ${\text{error}}_{\text{target}}$, adjusting $N_{\text{elas}}$ and $N_{\text{base}}$ is required. The inverse resolution process for a simple curve example is described in detail in the Supplementary Materials (Section S4).

### Experimental validation and parametric analysis

**Fig. 4.**
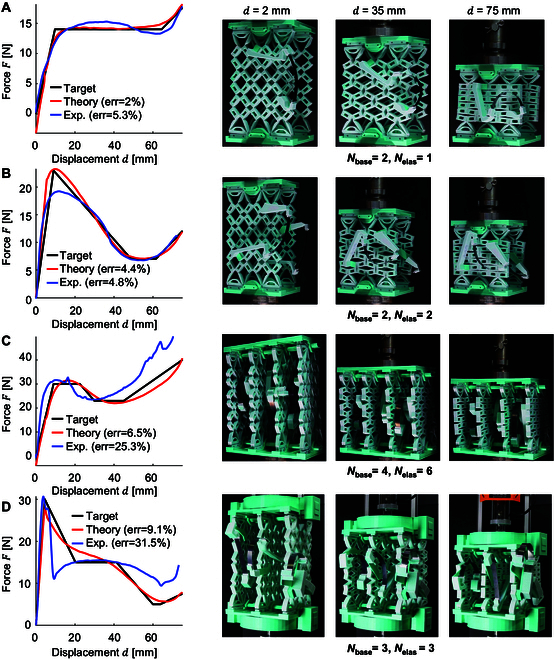
The experimental results of the inverse design for 1DOFmats are based on 4 different target responses. (A) Zero stiffness. (B) Large negative stiffness. (C) Two plateaus at different values. (D) Multi-stage softening. On the right are experimental snapshots taken at load displacements of *d* = 2 mm, *d* = 35 mm, and *d* = 75 mm, where $N_{\text{base}}$ represents the total number of 1-DOF kinematic bases and $N_{\text{elas}}$ represents the total number of elastic components.

The proposed 1DOFmat integrates a 1-DOF kinematic base with elastic components, with each component attached to the base at 2 end points. The base features 36 independently rotating rectangular blocks, enabling these end points to provide the elastic components with extensive motion flexibility. This configuration allows the coupled system to exhibit a variety of motion behaviors, facilitating the realization of arbitrarily stress–strain curves (see Movie S3 and Code S2). To verify the generalizability of the 1DOFmat, 4 different force–displacement curves were generated. Each target curve included 10 sample points (Section S4), and errors were calculated by comparing the inverse solution theory curve and the experimental test curve with the target curve, as shown in Fig. 4*.* The black, red, and blue lines represent the target curve, the inverse solution curve, and the experimental test curve, respectively. $N_{\text{base}}$ and $N_{\text{elas}}$ represent the number of kinematic bases and elastic components. The left side of Fig. 4 shows the force curves, while the right side presents snapshots of the experimental compression tests.

Firstly, the first target curve represents a common force curve for zero stiffness, which can be effectively achieved with a single elastic component. We formulate a force design using an elastic component and one kinematic base ($N_{\text{base}}$ = 2, $N_{\text{elas}}$ = 1). The designed response proposed by the 1DOFmat model closely approximates the target response, resulting in an error of 2%, while the experimental fitting error is also very accurate at 5.3%. The zero stiffness curve demonstrates marked potential for various applications in frequency vibration reduction, where effective control of vibrations across a wide range of frequencies is crucial to enhancing performance and ensuring structural integrity.

Second, we considered a target response with a large negative stiffness curve ($N_{\text{base}}$ = 2, $N_{\text{elas}}$ = 2), where the stiffness suddenly changes from positive to negative. The material can rapidly shift from “rigid” to “flexible”, making it suitable for applications that require rapid adjustments in stiffness. The matched error for the 1DOFmat-designed response is 4.4%, while the experimental fitting error is also low at 4.8%.

Third, we explore the design of a double gradient response curve ($N_{\text{base}}$ = 4, $N_{\text{elas}}$ = 6), where the force is initially 30 N and transitions to 20 N in the second stage. This can be used in scenarios where multiple gradient switches are required for different load requirements. The optimized solution of the 1DOFmat model aligns closely with the target response curve, with an error of 6.5%, while the experimental adjustment error is 25.3%.

Fourth, we considered a multistage softening response ($N_{\text{base}}$ = 3, $N_{\text{elas}}$ = 3), representing a curve of gradual softening. This type of response addresses a limitation where multi-DOF deformable metamaterials cannot be achieved. The error for the inverse solution of 1DOFmat is 9.1%, while the experimental fitting error is 31.5%. Due to the large energy variation required by multistage softening, the synchronization constraints on the base need to be improved, placing greater demands on the stiffness and torsional resistance of the base hinges.

**Fig. 5.**
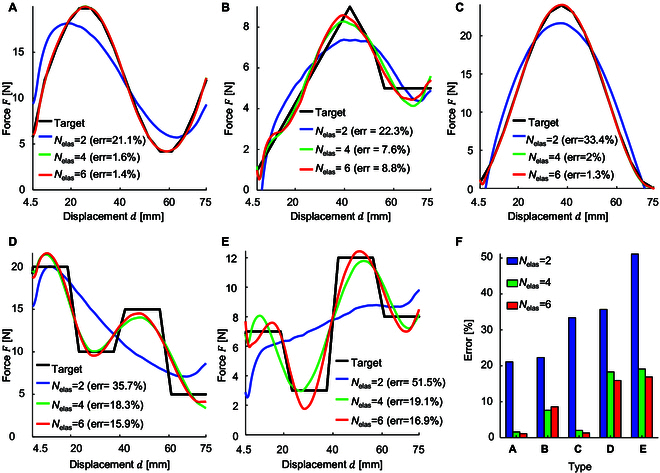
Parameter theory study on the number of elastic components. (A to E) Inverse designs for 5 target force–displacement curves, where $N_{\text{elas}}$ denotes the total number of elastic components. (F) Relationship between the quantity of elastic components and the fitting error.

We studied the relationship between the number of elastic components and the fitting error of the target curve, fixing the number of kinematic bases ($N_{\text{base}}$ = 2) and ensuring that the base reactions are consistent. The base configuration can be diversified, ensuring that the kinematic base retains a one degree of freedom. Figure 5 shows 5 target curves fits obtained by adjusting the number of elastic components; Fig. 5A to C shows a sinusoidal curve, a linear softening curve, and a cosine curve; Fig. 5D and E displays 2 distinct response curves with variations in peak and valley values. These 5 distinct target response curves illustrate the versatility of the 1DOFmat model in fitting complex response curves. Figure 5F shows the fitting errors for these target curves with different elastic components ($N_{\text{elas}}$ = 2, $N_{\text{elas}}$ = 4, $N_{\text{elas}}$ = 6), demonstrating that increasing the number of elastic components improves the fitting of the force–displacement curves, particularly when intricate stiffness variations are involved (see Code S3).

### Demonstration of rapid reprogramming capabilities and anisotropic designability

Active mechanical metamaterials and the active control of stimuli-responsive materials have garnered substantial research interest due to their potential for dynamic reprogrammability. The physical reprogrammability of pneumatically driven metamaterials typically requires exceptional sealing performance and a sophisticated pneumatic drive system [48,61,62]. Several approaches to reprogrammability are available, including SMA actuators, magnetic actuators, electromagnetic switches, and pneumatic actuators, each offering distinct advantages in terms of adaptability and control (see Section S1.3). In this study, choosing SMA allows for rapid reconfiguration of performance in response to external stimuli with minimal alteration, enabling the metamaterials to achieve enhanced properties.

**Fig. 6.**
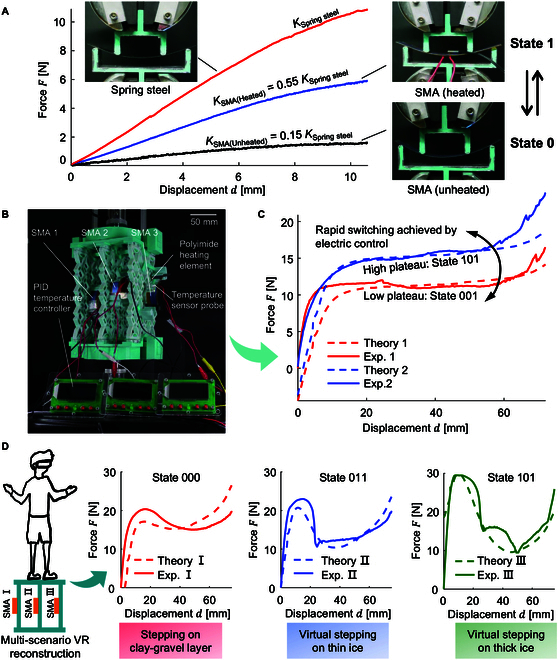
Preliminary demonstration of in situ rapid reprogramming. (A) Four-point bending test comparing the stiffness of spring steel and SMAs before and after heating. (B) Experimental setup demonstrating rapid reconfiguration using SMA as a replacement for spring steel. (C) Experimental curves of a quasi-zero stiffness vibration isolator with 2 different plateaus. (D) Preliminary demonstration of simulating stepping on clay–gravel layer [63], thin ice [64], and thick ice [64].

To compare the stiffness of the SMAs before and after heating with traditional spring steel, a 4-point bending test was performed, the specimen dimensions being 100 mm in length, 15 mm in width, and 0.3 mm in thickness (see Fig. 6A). The selected transition temperature for the SMA is 70 °C. When not heated, the SMA remains in the martensite phase at low temperatures, showing a “soft phase”, while upon heating it transitions to the austenite phase at high temperatures, showing a “stiff phase”. SMAs exhibit superelastic behavior at high temperatures in their austenitic state. The stiffness of heated SMA is 0.55 times of the spring steel chosen before; the stiffness of unheated SMA is 0.15 times of the spring steel. In the in situ rapid reconfigurable metamaterial experiment, a polyimide heating element was used to heat the SMA sheet, with a temperature sensor probe attached to its surface (Fig. 6B). A proportional-integral-derivative (PID) temperature controller, a feedback control system that continuously adjusts the control input based on the error between the setpoint and the measured process variable, was used to ensure that the SMA remained above its transition temperature of 70 °C, thereby maintaining the high stiffness characteristic of the stiff phase (see Movie S4).

The first experiment illustrates an example of “in situ rapid activation”, where a quasi-zero-stiffness vibration isolator adapts to varying loads with different plateaus. To meet diverse load requirements, the designed metamaterial features switchable force–displacement curves with tunable plateaus. Figure 6C displays the rapidly reconfigurable metamaterial consisting of 3 layers of SMA, where “0” denotes the soft state of the unheated SMA and “1” indicates the stiff state of the heated SMA. In the “001” state, the material shows a low load vibration reduction effect shown in red, while in the “101” state, it exhibits a high load vibration reduction effect depicted in blue. Furthermore, by modifying the size and number of SMA sheets, a wider range of target curves can be inversely designed, offering more options for various operating conditions and application scenarios. Figure 6D presents another set of rapid reconfiguration examples that used the same base and SMA sheets but designed different base-coupled components, resulting in 3 distinct response curves: a quasi-zero stiffness curve, a small-amplitude negative stiffness curve, and a large-amplitude negative stiffness curve. These innovative designs are particularly relevant for applications in virtual reality (VR) haptics, where they can effectively simulate various surface conditions, including clay–gravel layer [63], thin ice, and thick ice. By accurately replicating the tactile feedback associated with these different terrains, the proposed metamaterials enhance the immersive experience of VR environments. This advancement opens new avenues for VR applications, particularly in enhancing footstep interactions and providing users with a more realistic and engaging sensory experience [63–65]. Furthermore, these variations illustrate the flexibility and adaptability of the metamaterial design, allowing for tailored performance characteristics suited to specific applications. Beyond VR, this technology could be utilized in various fields such as soft robotics, adaptive structures, and biomechanical systems. One promising application is the development of variable stiffness materials that can actively change their rigidity in response to external stimuli, which could be used in impact-resistant materials, shock absorbers, or exoskeletons. For example, 3D polycatenated architected materials, which are designed with intricate, interconnected geometric structures, can enable these materials to switch between multiple stable states, offering a range of adjustable mechanical properties suitable for a variety of applications, including self-assembling systems, deployable structures, or even energy-efficient buildings.

**Fig. 7.**
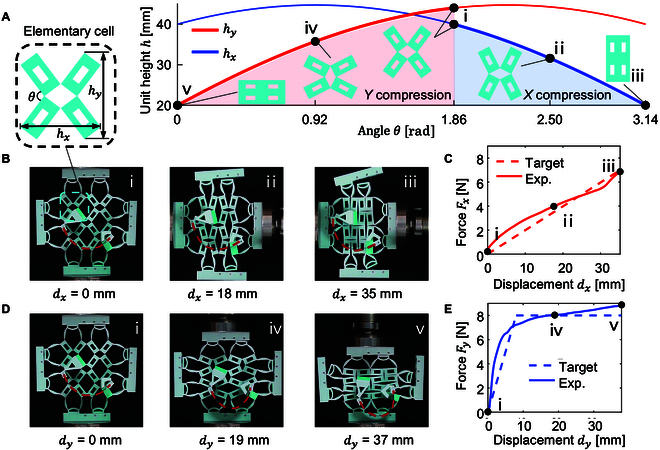
Preliminary overview of the anisotropic designability. (A) Relationship between the rectangular angle (θ) and the unit height (h) during compression in the *X* and *Y* directions. (B) Snapshots of the anisotropic metamaterial in 3 states of *X* compression. (C) Force–displacement curves for compression along the *X* direction. (D) Snapshots of the anisotropic metamaterial in 3 states of *Y* compression. (E) Force–displacement curves for compression along the *Y* direction.

The 1DOFmat model is capable of designing the force response not only in the *Y* direction but also in the *X* direction. Figure 7A illustrates the variation of the rectangular angle θ and the unit height h during compression in the *X* and *Y* directions. State i is the initial state (θ = 1.86), states ii and iii indicate compression in the *X* direction, and states iv and v represent compression in the *Y* direction. The *X*-direction compression curve (Fig. 7B and C) exhibits a nearly linear force response with an elastic beam span changing very slightly, while the *Y*-direction compression curve (Fig. 7D and E) transitions from high stiffness to zero stiffness with a substantially reduced elastic beam span. Due to the different paths of the angle θ changes corresponding to the compression of the *X* direction and the *Y* direction, representing different rotation modes of the rectangular cell, 2 distinct force curves can be designed (see Movie S5). Anisotropic responses can be arbitrarily customized, with distinct force–displacement curves in the *X* and *Y* directions, as discussed in Section S4.3. Unlike the design of force–displacement curves in a single direction, achieving anisotropic responses requires 2 different curves with the same set of elastic components. This increases the number of target curves for inverse design, necessitating more design parameters and computational resources. Furthermore, anisotropic responses can be made reconfigurable; by replacing the spring steels with smart materials, rapid reconfiguration of these responses can be achieved.

## Discussion

We introduced the 1DOFmat platform, which combines 1-DOF kinematic bases with elastic components. This method allows for the design of metamaterial stress–strain curves that can be customized to achieve various properties, ranging from single-gradient curves to large negative stiffness curves and multistage stress-softening curves. We studied the force curves of linear and nonlinear units connected in series and discovered that metamaterials with multi-DOF deformation modes cannot achieve multi-stage strain softening curves because of the minimum energy gradient principle. Experimental verification confirmed the effectiveness of the 1DOFmat in solving 4 different target force curves. A parametric study also analyzed the influence of the number of elastic components on the fitting errors of 5 distinct force curves. Finally, SMAs were introduced to enable in situ rapid reprogramming and the creation of anisotropic force curves. The proposed 1DOFmat framework is adaptable to a broader range of related fields, offering a new approach to the design of arbitrary mechanical metamaterials. The capabilities of in situ rapid reprogramming and anisotropic force curves can be utilized in applications such as adaptive structural systems, soft robotics, and advanced haptic interfaces, enhancing performance and responsiveness in dynamic environments. The capabilities of in situ rapid reprogramming and anisotropic force curves can be utilized in applications such as adaptive structural systems, soft robotics, and advanced haptic interfaces, enhancing performance and responsiveness in dynamic environments.

## Methods

Information about the methods used in this research is available in the Supplementary Material.

## Data Availability

All data generated or analyzed during this study are included in the text content or Supplementary Materials of this published article.

## Supplementary Materials

Supplementary 1 Sections S1 to S5 Figs. S1 to S11 Tables S1 and S2 Movies S1 to S5 Codes S1 to S6
