# Supplementary material for: One-Degree-of-Freedom Mechanical Metamaterials with Arbitrary Prescribability and Rapid Reprogrammability of Force–Displacement Curves
Source: Research (Wash D C). 2025 Jun 9;8:0715. doi: 10.34133/research.0715 (PMC12136626; doi:10.34133/research.0715)
Supplement: Supplementary 1 — Sections S1 to S5 Figs. S1 to S11 Tables S1 and S2 Movies S1 to S5 Codes S1 to S6 [file research.0715.f1.zip › 02_Supplementary_information.pdf]

Supporting Information
One-DOF Mechanical Metamaterials with Arbitrary Prescribability and Rapid Reprogrammability of Force-Displacement Curves

*Hui Li, Wei Li, Huixin Yang, Joseph M. Gattas, Qingyang Chen, Yang Li**

H. Li, Q. Chen, Y. Li
The Institute of Technological Sciences, Wuhan University; Wuhan, Hubei, 430072, China
E-mail: yang.li@whu.edu.cn

W. Li
Hongyi Honor College, Wuhan University; Wuhan, Hubei, 430072, China

H. Yang
School of Power and Mechanical Engineering, Wuhan University, Wuhan, Hubei, 430072, China

J.M. Gattas
School of Civil Engineering, The University of Queensland, Brisbane, 4072, Australia

Y. Li
Wuhan University Shenzhen Research Institute, Shenzhen, 518063,China

Contents

S1 Physical platform **2**
S1.1 Our physical platform . . . 2
S1.2 Exploration of 1-DOF kinematic bases . . . 4
S1.3 Comparison of reprogrammable metamaterial actuators . . . 6

S2 Fabrication and experiments **6**

S3 Interpretation for 2-DOF metamaterials **9**
S3.1 Force-displacement curve of two linear springs in series . . . 9
S3.2 Force-displacement curve of two nonlinear units in series . . . 10

S4 Introductory cases **11**
S4.1 Inverse design process . . . 11
S4.2 A simple example of the inverse design . . . 14
S4.3 Inverse design of anisotropic responses . . . 15

S5 Formulas block **17**

S1. Physical platform

S1.1 Our physical platform

Our experimental setup includes one-degree-of-freedom (1-DOF) kinematic bases with elastic components. This kinematic base consists of rectangular units, trapezoidal structures, a top frame, and a bottom frame (see Figure S1). The central rectangular blocks function as a scissor link, allowing free vertical sliding. The central trapezoidal structure is fixed to the frame, whereas the trapezoidal structures on the left and right sides can slide horizontally relative to the frame. This design provides a flexible and adaptable platform for exploring various kinematic behaviors and energy transformations.

The kinematic base has a length $H_0 = 200$ mm, a width $L = 117$ mm, and a thickness of 10 mm, each rectangular basic unit measures $a = 20$ mm in length and $b = 10$ mm in width, featuring a central rectangular through-hole of 10 mm by 5 mm. The initial angle between the upper and lower rectangular basic units is $2\alpha_0 = 106°$, the trapezoidal structure is connected to the rectangular basic unit on its short side, with a long base edge length $d = 37$ mm. The initial distance between its upper and lower edges is $f_0 = 22$ mm, with the waist at $c = 24.4$ mm and an initial angle between the bottom edge and the waist of $\beta = 64$ °. The distance from the trapezoidal structure to the frame is $g = 12$ mm. The initial distance between the upper and lower rectangular basics is $h_0 = 44$ mm.

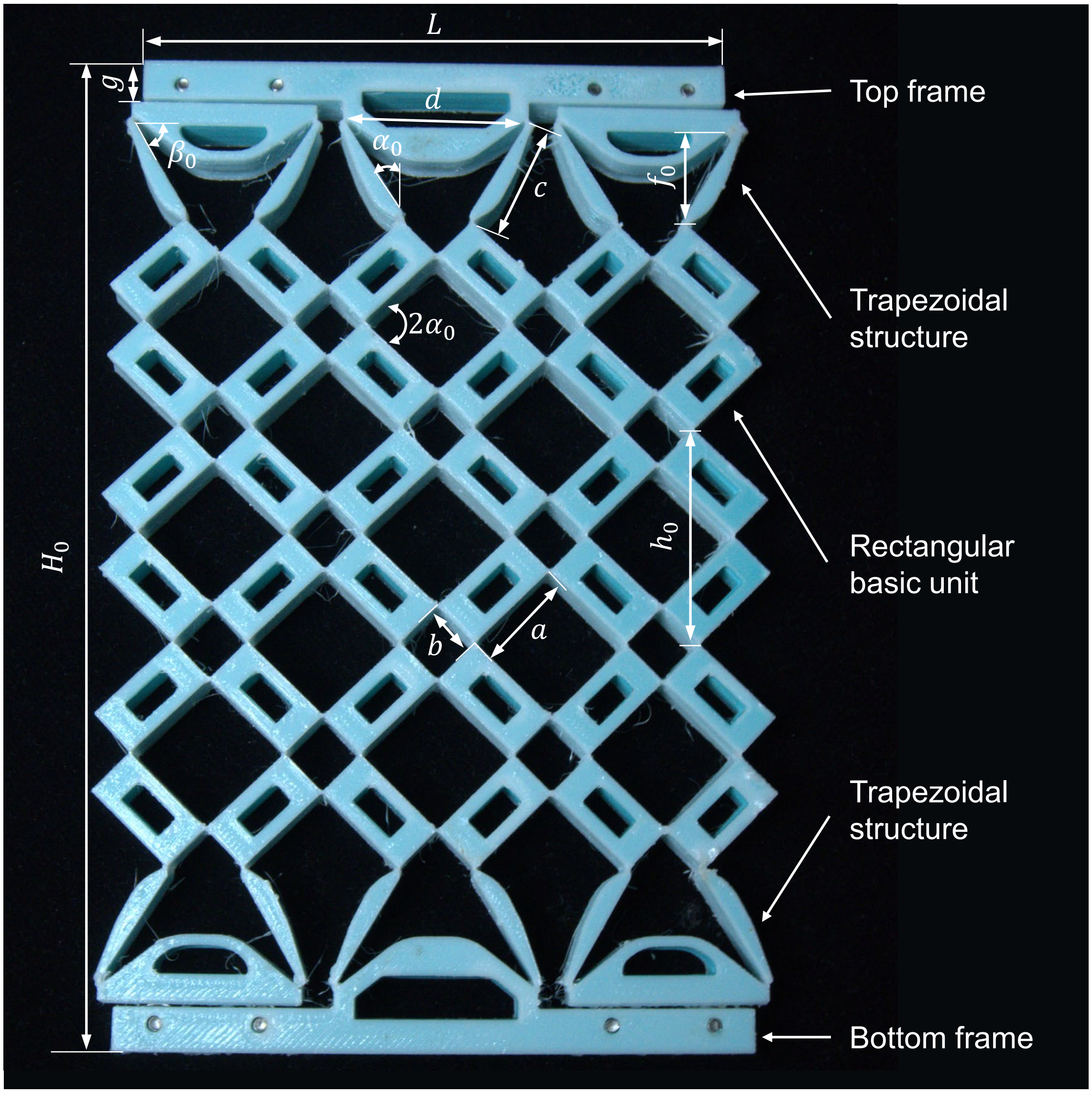

Figure S1. Schematics of the 1-DOF kinematic base

The unfolding interaction occurs as the 1-DOF kinematic base undergoes compression. In this mechanical system, each rectangular basic unit gracefully rotates, while the trapezoidal structures on both the left and right sides elegantly slide horizontally relative to the frame. This synchronized movement is a pivotal aspect of the system's functionality, enabling a responsive and harmonious interplay among the components during the compression process.

In the compression process, the kinematic base is divided into N_{test} sample points, with the compression displacement of each test point denoted by d^j_{disp}. The displacement d^j_{disp} is determined by subtracting the total length H^j at each test state from the initial length H_0. The total displacement H^j at each test point can be expressed in terms of the angle α^j between the upper and lower rectangular basic units. The length of the bottom edge d of the trapezoidal structure remains constant during the motion process, the relationship between α^j and β^j is established:

$$d^j_{disp} = H_0 - H^j, \tag{S1}$$

$$H^j = 6(a \sin \alpha^j + b \cos \alpha^j) + 2c \sin \beta^j + 2g, \tag{S2}$$

$$d = 2(b \sin \alpha^j + c \cos \beta^j), \tag{S3}$$

Solving the equations simultaneously determines the relationship between d^j_{disp} and α^j , with each rectangular basic unit defining a local coordinate system attached to the body (see Figure S2).

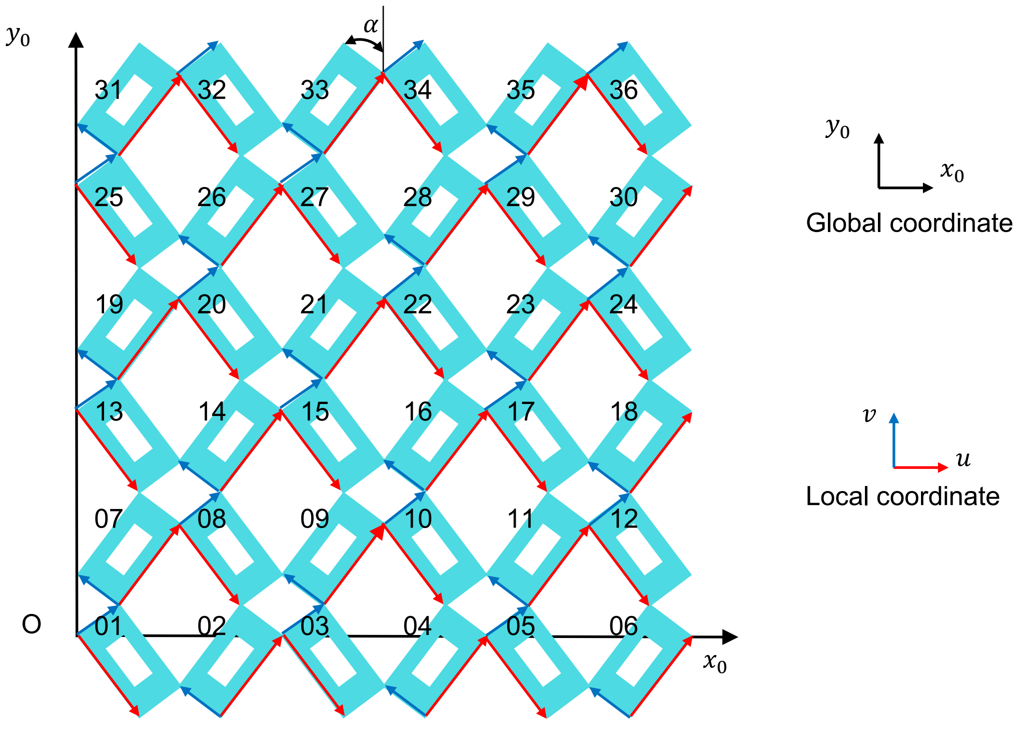

Figure S2. The identification and local coordinate systems of the 36 rectangular basic units.

Each 1-DOF kinematic base can be connected to at least two elastic components (positioned on the front and back), and the elastic components can be chosen from among the 36 rectangular basic units. Utilizing the theory of homogeneous coordinate transformation in robotics [1], the position point of the elastic component connecting the spring steel is transformed from the local coordinate system $p(u, v)$ to the global coordinate system $P(x, y)$, the distance l^i_s between the two endpoints of the spring steel in the elastic components during compression is expressed:

$$l^i_s = \sqrt{(x^i_1 - x^i_2)^2 + (y^i_1 - y^i_2)^2}, \tag{S4}$$

Table S1. Designs for all design variables considered in the main text.

Designs	N_{elas}	N_1	N_2	u_1	v_1	u_2	v_2	L_0	w
Fig.3B Positive	1	4	34	10	5	10	5	115	10
Fig.3B Negative	1	15	33	29.553	30.438	50	-50	115	10
Fig.4A	1	33	12	44.283	-45.51	25.143	8.804	96.056	6.381
Fig.4B	2	24	4	-41.203	-46.946	-33.924	42.969	94.042	8.000
		2	29	-13.13	49.489	-23.01	-49.796	98.142	7.958
Fig.4C	6	16	30	-17.074	-3.6918	-18.492	30.348	93.01	11.502
		21	32	-27.8	-33.2	49.98	-49.96	93.055	12.534
		9	14	23.03	-22.24	-14.31	-2.44	93.096	12.498
		26	27	32.021	49.328	-38.72	-32.783	93.000	12.543
		35	8	32.599	-39.489	43.01	59.796	92.984	12.502
		17	23	-44.967	-8.5104	-27.179	-40.116	92.903	12.491
Fig.4D	3	15	18	57.342	35.768	74.607	73.397	95.527	10.761
		20	16	-20.944	-43.637	-57.242	58.513	98.055	11.934
		4	29	-13.887	-6.721	49.310	-16.589	98.096	10.898
Fig.6C	3	9	19	-22.677	16.961	-5.025	6.984	100	15
		14	34	-5.409	49.890	-15.642	-44.170	100	15
		8	33	26.721	26.589	33.887	-39.310	100	15
Fig.6D	3	20	9	-49.257	-49.653	-55.409	34.190	100	15
		2	23	9.772	-37.381	-14.097	36.095	100	15
		9	32	-50.328	9.263	60.000	-60.000	100	15

where i is the identification number of the elastic components, P1(x_1^i, y_1^i) and P2(x_2^i, y_2^i) are the global coordinates of the two endpoints of the spring steel. The design variables for all geometries considered in the main text are reported in Table S1.

S1.2 Exploration of 1-DOF kinematic bases

Beyond our proposed physical platform, a variety of unit cell, such as triangle, square, rectangle, parallelogram and irregular quadrilateral, illustrated in Figure S3. The key requirement is that the chosen kinematic base must be one degree of freedom. Achieving arbitrary force-displacement curves through inverse design requires an increasing number of design variables, which allow for more complex force profiles. As the design variables of the kinematic base expand, the kinematics become more diverse, enabling a broader range of nonlinear motions. These nonlinear deformations facilitate intricate energy transformations, leading to more sophisticated force-displacement curves. Another critical factor to consider are boundary contacts. Given that conventional metamaterial applications often involve tension or compression, a higher number of boundary contacts improves the suitability of the material for practical applications.

Selecting different topological connections, along with unit cells for kinematic structures, enables a wide range of kinematics, providing a flexible approach to designing kinematic bases with diverse behaviors, as illustrated in Figure S4. In addition to two-dimensional kinematic bases, three-dimensional configurations present new possibilities. The scissor beam coupled with Sarrus linkages facilitates the realization of a 3D one-degree-of-freedom kinematic base. The third dimension introduces an extra degree of motion, broadening the scope for energy transformations. Kinematic bases with more design variables and boundary contacts are increasingly preferred as metamaterial foundations, offering enhanced versatility and improving the practicality of applications.

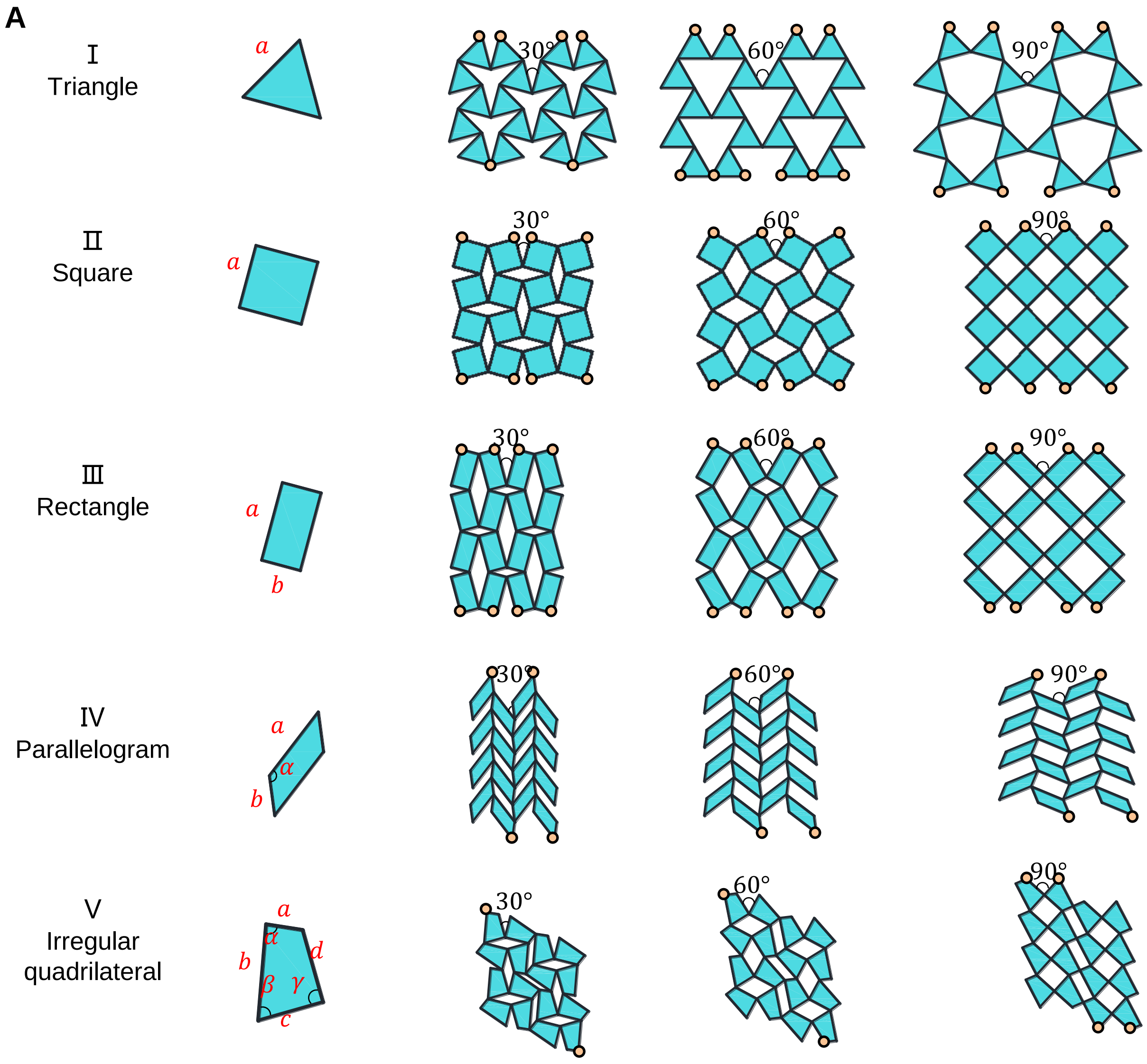

B

Category	I Triangle	II Square	III Rectangle	IV Parallelogram	V Irregular quadrilateral
Number of degrees of freedom	4	1	1	1	1
Number of design variables	1	1	2	3	7
Minimum number of boundary contacts	2	4	4	2	1

Figure S3. Exploring different unit cells for kinematic structures. (**A**) Schematic of deformations for five different unit cells. (**B**) Comparison of five types of unit cells based on their number of degrees of freedom, number of design variables, and minimum number of boundary contacts.

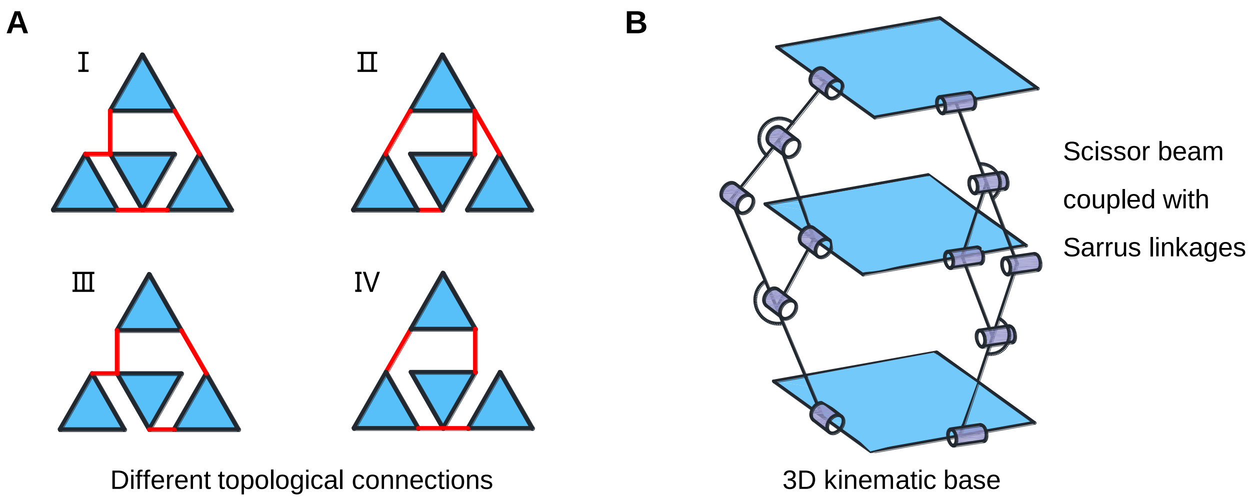

Figure S4. Different topological connections and 3D kinematic structures. (**A**) Various topological connections with unit cells for kinematic structures. (**B**) A primarily three-dimensional 1-DOF kinematic base prototype.

S1.3 Comparison of reprogrammable metamaterial actuators

Metamaterials with real-time active changes in mechanical properties can be broadly classified into two categories. The first category involves changing the structural geometry of the metamaterial through mechanical deformation, thereby altering its mechanical response. The second approach modifies the metamaterial using smart materials. Compared to motors and mechanical drives, electrically controlled smart materials are more lightweight. A comparison of these reprogramming methods based on key parameters, including the strength-to-weight ratio, the power source, the size, the stiffness control, the response time, the wireless capability, and the environmental sensitivity, is presented in Table S2.

Table S2. Comparison of different actuators.

textbfProperty	**SMA actuators**	**Magnetic actuators**	**Electromagnetic switches**	**Pneumatic**
Strength-to-weight ratio	High	Moderate	Low	Moderate
Power source	Electrical	Electrical	Electrical	Compressed air
Size	Modest	Compact	Compact	Generally larger
Stiffness control	Active variable stiffness	Limited stiffness control	Switches between two states (on/off)	Variable stiffness limited to air pressure
Response time	Moderate	Fast (instantaneous switching)	Very fast (instant switching)	Fast
Wireless	Feasibly	Easily	Feasibly	Difficultly
Environmental sensitivity	Sensitive to temperature	Affected by magnetic field interference	Not significantly affected	Sensitive to airtightness

S2. Fabrication and experiments

Preliminary view of the physical metamaterial, as shown in Figure S5. The metamaterial is fabricated using the dual-material printer Ultimaker S5, employing two materials with different elastic moduli: soft TPU for the flexible hinge and rigid PLA for the supporting plane structure. The elastic component and 1-DOF kinematic base are connected via a connector pin, with rectangular through-holes at different positions to enable selectable kinematics of the elastic component's two endpoints. The spring steel is

wrapped with a TPU spacer and inserted into the flexible hinge, enabling free rotation relative to the two endpoints of the elastic component, thus achieving different elastic properties.

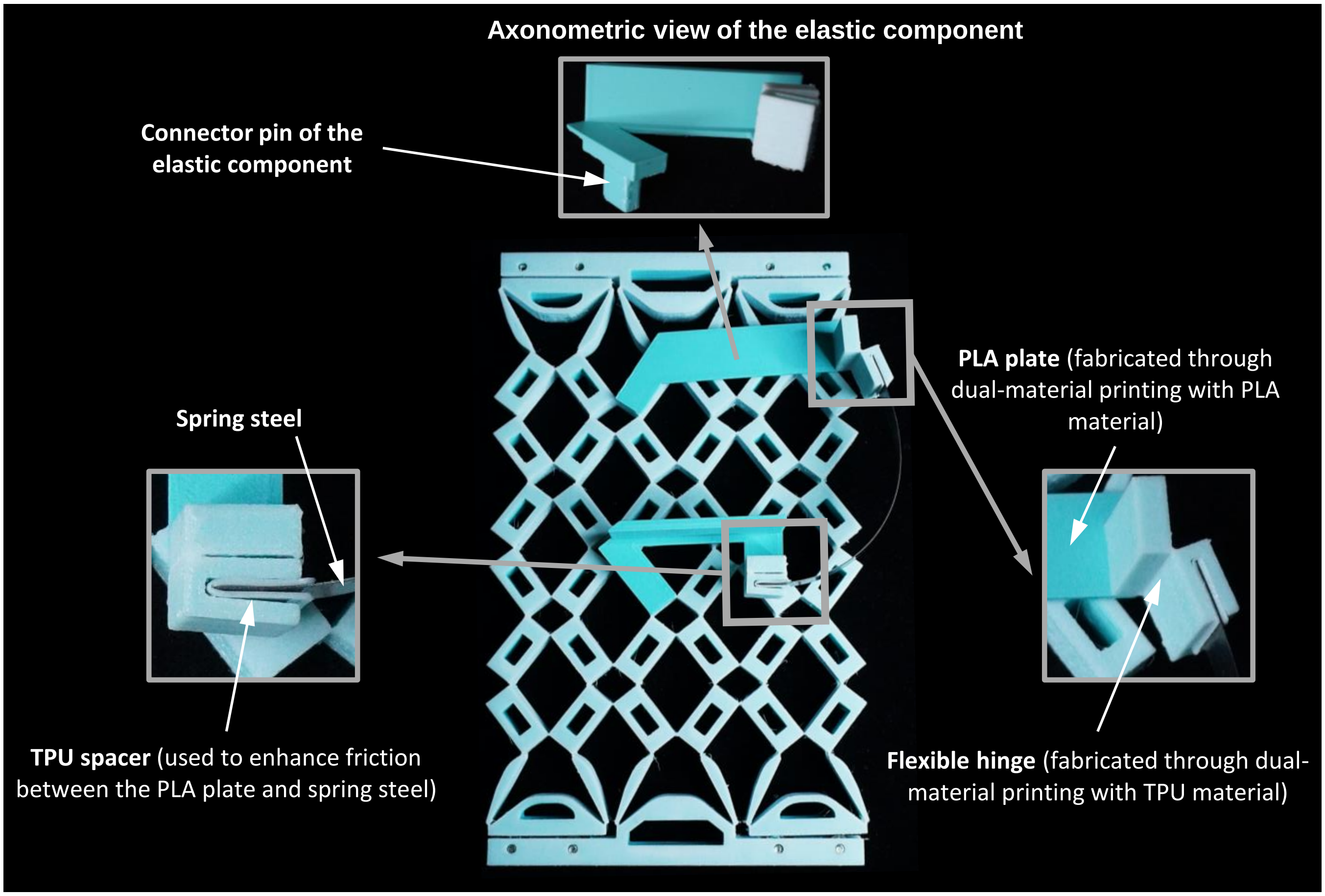

Figure S5. Physical snapshot of the metamaterial and an overview of the approaches for connecting different components.

Kinematic bases and elastic components are modeled using SolidWorks, and the physical model is 3D printed with the Ultimaker S5, as shown in Figure S6. The 1-DOF kinematic base is printed with thermoplastic polyurethane (TPU) wrapped around polylactic acid (PLA), the flexible hinges connecting the rectangular basic units and the trapezoidal structure are made of TPU with dimensions of 10 mm in length, 1 mm in width, and 0.4 mm in thickness.

Each elastic component consists of two elastomeric couplers (inserted into the through holes of the rectangular basic units) and a spring steel. To minimize friction between the trapezoidal structure and the upper and lower frame frames during lateral movement, each upper and lower frame is equipped with four screws to prevent frictional force during motion from affecting the response of the metamaterial (see Figure S6*A*). As the number of kinematic bases increases, connecting rods are employed to link the top frame and bottom frame of the bases through connecting plates. This configuration ensures that the compression displacement of each base remains uniform during the compression process, facilitating synchronized motion among the kinematic bases (see Figure S6*B*).

The reactive forces of the kinematic bases are tested using the MTS Systems Corporation model C43.104. Due to the viscoelastic properties of the 3D printed TPU material, the compressed structure does not fully return to its original printed position after multiple compressions, resulting in zero point drift. To validate the error between theoretical calculations of the ECKBM model and experimental results, the initial position is first calibrated during the testing of the reactive forces of the 1-DOF kinematic base (see Figure S7*A*). Subsequently, the tensile force exerted by the 1-DOF kinematic base as it returns to the initial printed position after zero-point drift is under examination (see Figure S7*B*), along with the compressive force from zero-point drift to the end of compression (see Figure S7*C*-*E*). The force curve obtained from experimental testing on two 1-DOF kinematic bases is shown in Figure S8.

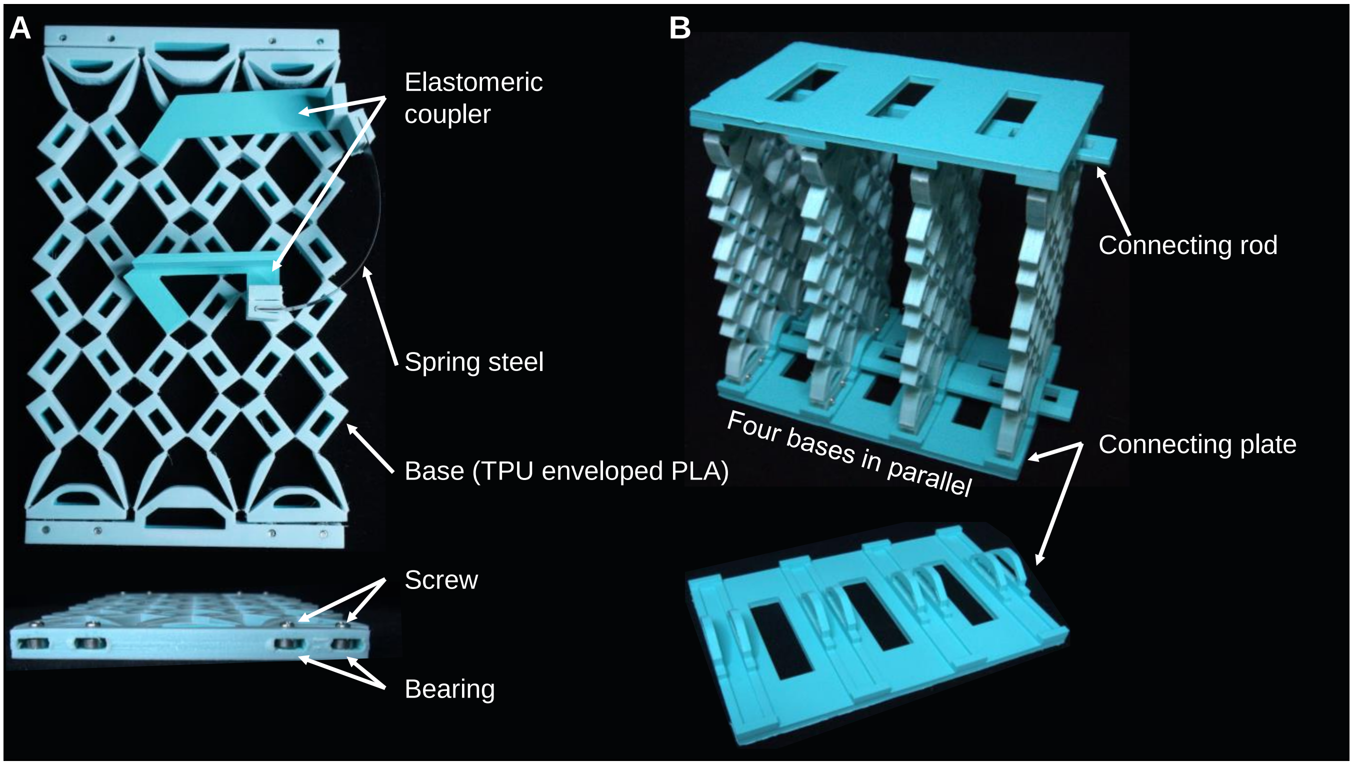

Figure S6. Fabrication and assembly. (**A**) Schematic diagram of one elastic component connected to a 1-DOF kinematic base. (**B**) Four 1-DOF kinematic bases are connected in parallel.

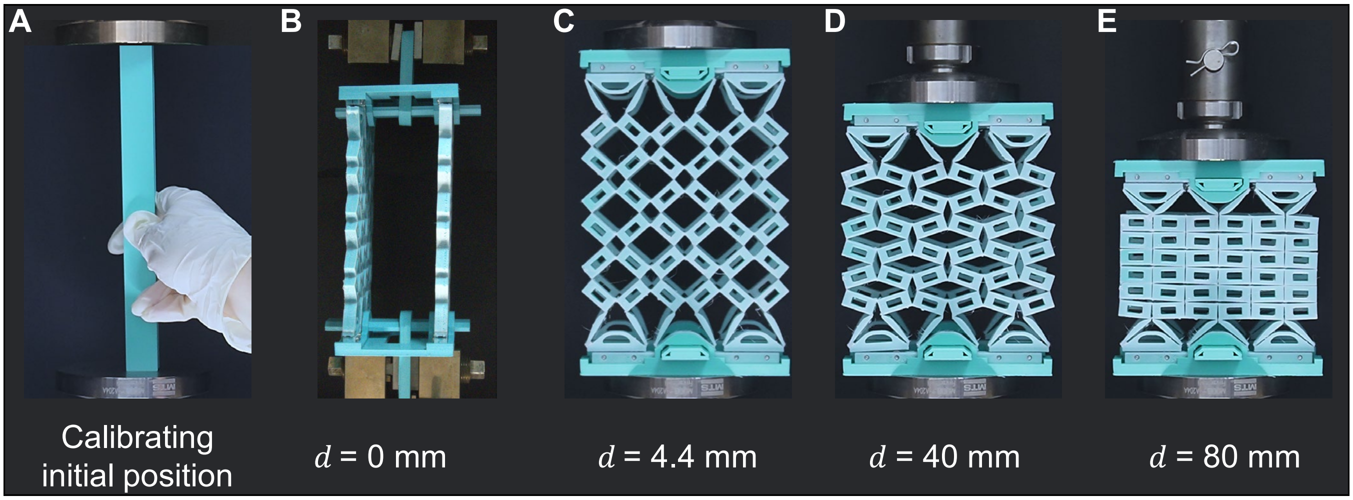

Figure S7. Reaction force testing of 1-DOF kinematic bases. (**A**) Calibrating the initial position. (**B**) Snapshot of the tension test at the initial position. (**C-E**) Snapshots of the 1-DOF kinematic bases compression test at displacements of d = 4.4 mm, d = 40 mm, and d = 80 mm.

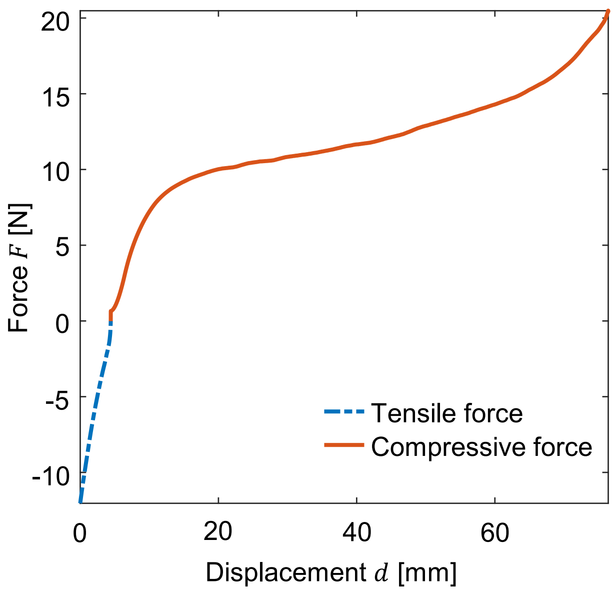

Figure S8. The force-displacement test curve of the 1-DOF kinematic bases.

S3. Interpretation for 2-DOF metamaterials

S3.1 Force-displacement curve of two linear springs in series

Two linear springs with different stiffness ($k_{linear}^{soft} = 0.1\,\mathrm{N\,mm^{-1}}, k_{linear}^{stiff} = 0.2\,\mathrm{N\,mm^{-1}}$), both compressed with a limit displacement of 10 mm are connected in series, the sum of the deformations of the two linear springs equals the total displacement of the material:

$$d_{linear}^{2DOF} = \Delta l_{linear}^{soft} + \Delta l_{linear}^{stiff}, \tag{S5}$$

the energy during the compression process can be expressed by the deformation of two linear springs:

$$E_{linear}^{total} = \frac{1}{2} k_{linear}^{soft} \, {\Delta l_{linear}^{soft}}^{2} + \frac{1}{2} k_{linear}^{stiff} \, {\Delta l_{linear}^{stiff}}^{2}, \tag{S6}$$

the principle of minimum energy gradient for multi-DOF systems suggests that the energy of a series linear elastic system tends towards its minimum point. This problem can be transformed into solving a constrained minimization problem, the Lagrange Multiplier Method is employed for this purpose [2]. The Lagrangian $\mathcal{L}$ is formulated as follows:

$$\begin{aligned} \text{Lagrangian:} \quad & \mathcal{L} = f + \lambda g, \\ \text{Objective:} \quad & f = \frac{1}{2} k_{linear}^{soft} \, {\Delta l_{linear}^{soft}}^{2} + \frac{1}{2} k_{linear}^{stiff} \, {\Delta l_{linear}^{stiff}}^{2}, \\ \text{Constraint:} \quad & g = \Delta l_{linear}^{soft} + \Delta l_{linear}^{stiff} - d_{linear}^{2DOF}, \end{aligned} \tag{S7}$$

where λ is the Lagrange multiplier. To solve the problem, we take partial derivatives of the variables and the Lagrange multiplier and set them equal to zero:

$$\begin{aligned} \frac{\partial \mathcal{L}}{\partial \Delta l_{linear}^{soft}} &= k_{linear}^{soft} \Delta l_{linear}^{soft} + \lambda = 0, \\ \frac{\partial \mathcal{L}}{\partial \Delta l_{linear}^{stiff}} &= k_{linear}^{stiff} \Delta l_{linear}^{stiff} + \lambda = 0, \\ \frac{\partial \mathcal{L}}{\partial \lambda} &= \Delta l_{linear}^{soft} + \Delta l_{linear}^{stiff} - d_{linear}^{2DOF} = 0, \end{aligned} \tag{S8}$$

when the stiffness values for the linear springs($k_{linear}^{soft} = 0.1\,\mathrm{N\,mm^{-1}}$, $k_{linear}^{stiff} = 0.2\,\mathrm{N\,mm^{-1}}$) are substituted into the above equations, the objective function is found to reach its minimum with $\Delta l_{linear}^{soft} = \frac{2}{3}d_{linear}^{2DOF}$, $\Delta l_{linear}^{stiff} = \frac{1}{3}d_{linear}^{2DOF}$. when $d_{linear}^{2DOF} \leq 15$ mm, the minimum value calculated using the Lagrange multiplier can be chosen, the stiffness of the series linear spring system is $\frac{2}{3} \cdot k_{linear}^{soft}$ or $\frac{1}{3} \cdot k_{linear}^{stiff}$ ($k_{linear}^{2DOF} = 0.067\,\mathrm{N\,mm^{-1}}$). When $d_{linear}^{2DOF} > 15$ mm, the deformation of the soft spring reaches its compression limit of 10 mm, which means that the soft linear spring is fully compressed and subsequent compression only involves the stiff linear spring ($k_{linear}^{2DOF} = 0.2\,\mathrm{N\,mm^{-1}}$).

S3.2 Force-displacement curve of two nonlinear units in series

Two nonlinear units with different stiffness ($F_{nonlin}^{soft} = \sin\left(\frac{2\pi}{10}\Delta l_{nonlin}^{soft}\right), F_{nonlin}^{stiff} = 2\sin\left(\frac{2\pi}{10}\Delta l_{nonlin}^{stiff}\right)$), both compressed with a total displacement of 10 mm, are connected in series. The sum of the deformations of the two nonlinear units equals the total displacement of the material:

$$d_{nonlin}^{2DOF} = \Delta l_{nonlin}^{soft} + \Delta l_{nonlin}^{stiff}, \tag{S9}$$

the energy during the compression process can be obtained by integrating the deformation of the two nonlinear springs:

$$\begin{aligned} E_{nonlin}^{soft} &= -\cos\left(\frac{2\pi}{10}\Delta l_{nonlin}^{soft}\right)\frac{10}{2\pi} + \frac{10}{2\pi}, \\ E_{nonlin}^{stiff} &= -2\cos\left(\frac{2\pi}{10}\Delta l_{nonlin}^{stiff}\right)\frac{10}{2\pi} + \frac{20}{2\pi}, \end{aligned} \tag{S10}$$

the total energy of the series of nonlinear units can be expressed as:

$$E_{nonlin}^{total} = -\frac{5}{\pi}\left[\cos\left(\frac{\pi}{5}\Delta l_{nonlin}^{soft}\right) + 2\cos\left(\frac{\pi}{5}\Delta l_{nonlin}^{soft}\right) - 3\right], \tag{S11}$$

the energy of the series nonlinear elastic system tends to the lowest point in multi-DOF systems. Similarly, this problem can be transformed into a constrained minimization problem [2]. Lagrange Multiplier Method is used to solve the problem, where the objective function and equality constraint are combined to form the Lagrangian function $\mathcal{L}$:

$$\begin{aligned} \text{Lagrangian:} \quad & \mathcal{L} = f + \lambda g, \\ \text{Objective:} \quad & f = -\frac{5}{\pi}\left(\cos\left(\frac{\pi}{5}\Delta l_{nonlin}^{soft}\right) + 2\cos\left(\frac{\pi}{5}\Delta l_{nonlin}^{soft}\right) - 3\right), \\ \text{Constraint:} \quad & g = \Delta l_{nonlin}^{soft} + \Delta l_{nonlin}^{stiff} - d_{nonlin}^{2DOF}, \end{aligned} \tag{S12}$$

where λ is the Lagrange multiplier. Take partial derivatives with variables and multipliers, and set them equal to zero:

$$\begin{aligned} \frac{\partial \mathcal{L}}{\partial \Delta l_{nonlin}^{soft}} &= \sin\left(\frac{\pi}{5}\Delta l_{nonlin}^{soft}\right) + \lambda = 0, \\ \frac{\partial \mathcal{L}}{\partial \Delta l_{nonlin}^{stiff}} &= 2\sin\left(\frac{\pi}{5}\Delta l_{nonlin}^{stiff}\right) + \lambda = 0, \\ \frac{\partial \mathcal{L}}{\partial \lambda} &= \Delta l_{nonlin}^{soft} + \Delta l_{nonlin}^{stiff} - d_{nonlin}^{2DOF} = 0, \end{aligned} \tag{S13}$$

solving the equation $\sin\left(\frac{\pi}{5}\Delta l_{nonlin}^{soft}\right) - 2\sin\left(\frac{\pi}{5}\Delta l_{nonlin}^{stiff}\right) = 0$ for an analytical solution is challenging, the findpeaks function in MATLAB is used to obtain initial guesses for energy minima, utilizing the fsolve function to precisely solve for solutions satisfying the Lagrange function (see Movie S1 and Code S1 in the Supporting Information).

In the first stage $d_{nonlin}^{2DOF} \leq 5$ mm, a single minimum point exists in the feasible region, in the second stage 5 mm< $d_{nonlin}^{2DOF} < 15$ mm, two minima points emerge in the feasible region, the minimum energy

gradient principle governing multi-DOF systems ensures that the system efficiently reaches the nearest minimum in the second stage. In the third stage $d_{nonlin}^{2DOF} \geq 15$ mm, there exists only one minimum point in the feasible region. At $d_{nonlin}^{2DOF} = 15$ mm, where the system has only one energy minimum point, the multi-DOF system immediately snaps to this minimum point. The sudden decrease in system energy transforms into the kinetic energy of the nonlinear series unit, resulting in an immediate drop in reactive forces to zero. Metamaterials with deformation modes in multi-DOF systems must adhere to the principle of minimum energy gradient. This paper approaches the topic from an energy perspective, confirming the fact that series-connected elastic units cannot achieve a multistage stress-softening curve.

S4. Introductory cases

S4.1 Inverse design process

The 1DOFmat is made up of kinematic bases and elastic components. The custom target curve is equivalent to the sum of the base force curve and the reaction force curve of the elastic components (see Figure S9A). The 1-DO kinematic base provides the fundamental structure upon which the 1DOFmat is constructed (Figure S9B). The elastic components are securely attached to the kinematic bases. The span of the spring steel varies, which induces an energy shift within the system. Each elastic component (Figure S9C) is defined by two integer variables and six real variables:

$$X_p = [N_1, N_2, u_1, v_1, u_2, v_2, L_0, w], \tag{S14}$$

the 1DOFmat reverse design process (see Figure S10) starts by entering the target response F_{target}, sample points N_{test} and the allowable error $error_{target}$. The quantities of elastic components and 1-DOF kinematic bases are initially selected. Subsequently, the reaction forces F_{base} of the kinematic bases of the 1DOF are tested.

Describing the parameters of the elastic components, each component is characterized by two integer variables (N_1, N_2) and six real variables $(u_1, v_1, u_2, v_2, L_0, w)$. Under compression displacement load, coupled linkage kinematics on the 1-DOF mechanism can be determined through homogeneous coordinate transformations. The distance between the two endpoints of the spring steel is l_s^i, the compression deformation of each spring steel is calculated:

$$\Delta l^i = L_0^i - l_s^i, \tag{S15}$$

where L_0^i is the rest length of each spring steel. In the compression process of the 1DOFmat under displacement load, the ends of the spring steel can freely rotate. The reactive force P_{elas}^i for each spring steel element is calculated by integrating through the theory of rod buckling [3]:

$$P_{elas}^i = P_{cr}^i \left(1 + \frac{\Delta l^i}{2L_0^i}\right), \tag{S16}$$

where P_{cr}^i is the critical pressure of free boundary rod buckling $P_{cr} = \frac{\pi^2 EI}{L_0^2}$, L_0 is the rest length of the spring steel, Δl^i is the deformation of the spring steel, E is the Young's modulus of the elastic component material, $I = \frac{wt^3}{12}$ is the moment of inertia of the material, w is the width of the spring steel, and t is the thickness of the spring steel, the energy of the elastic component is calculated:

$$E_{elas}^i = P_{cr}^i \, \Delta l^i + \frac{P_{cr}^i \, \Delta {l^i}^2}{4L_0^i}, \tag{S17}$$

the sum of the reactive force F_{base} from the 1-DOF kinematic bases and the elastic potential energy change E_{elas}^{total} caused by the deformation of the elastic component, projected onto the displacement load d_{disp}, equals the reactive force of the 1DOFmat metamaterial $F_{1DOFmat}$:

$$F_{1DOFmat} = F_{base} + \frac{\mathrm{d}E_{elas}^{total}}{\mathrm{d}d_{disp}}, \tag{S18}$$

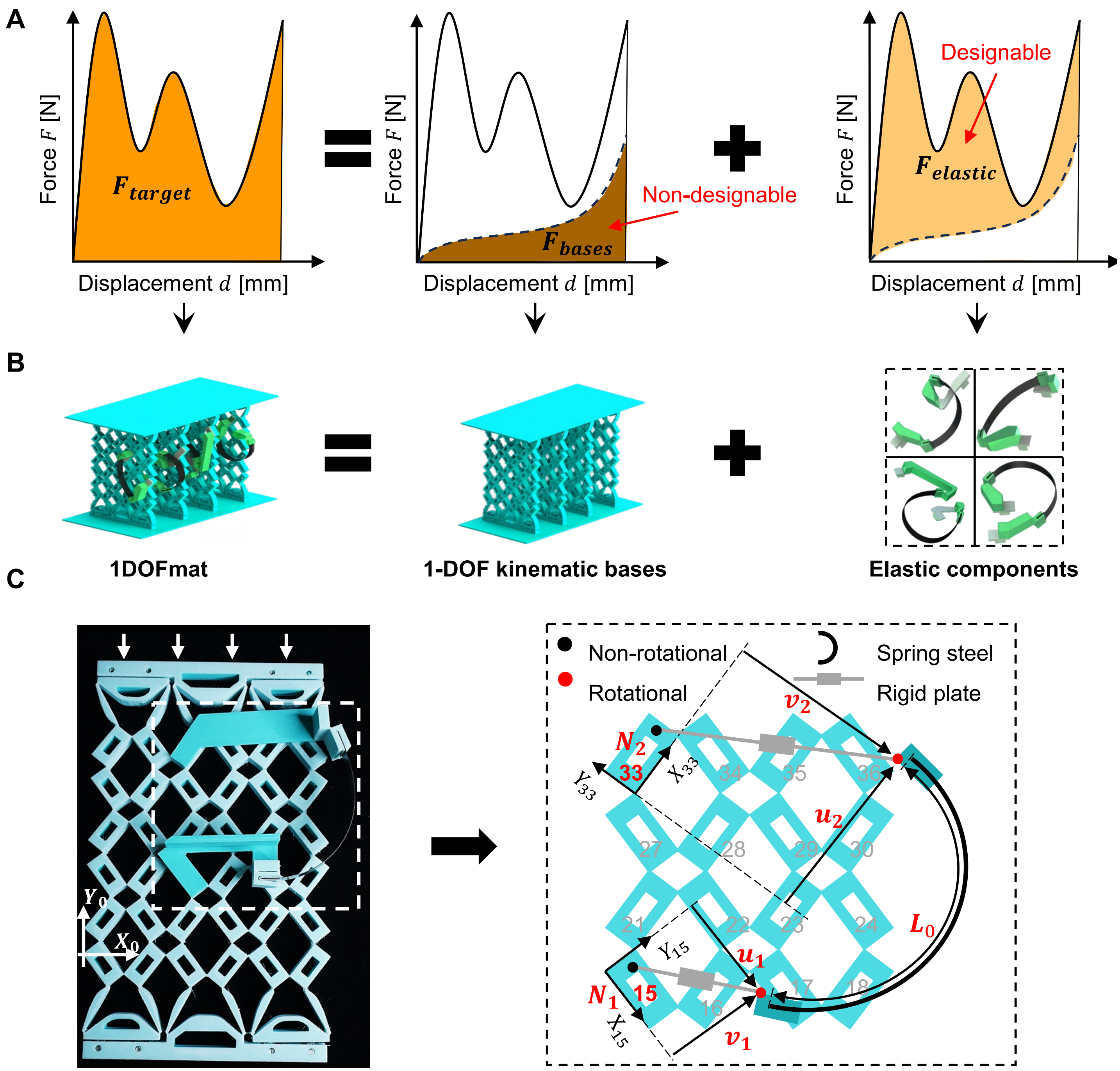

Figure S9. Schematic of 1DOFmat and parametric representation of elastic components. (**A**) The target response curve results from the combined responses of the 1-DOF kinematic base and the elastic components. (**B**) 1DOFmat comprises 1-DOF kinematic bases and elastic components, with various types of elastic components mounted to the kinematic bases. (**C**) The design parameters of the elastic components include two integer variables (N_1, N_2) and six real variables ($u_1, v_1, u_2, v_2, L_0, w$).

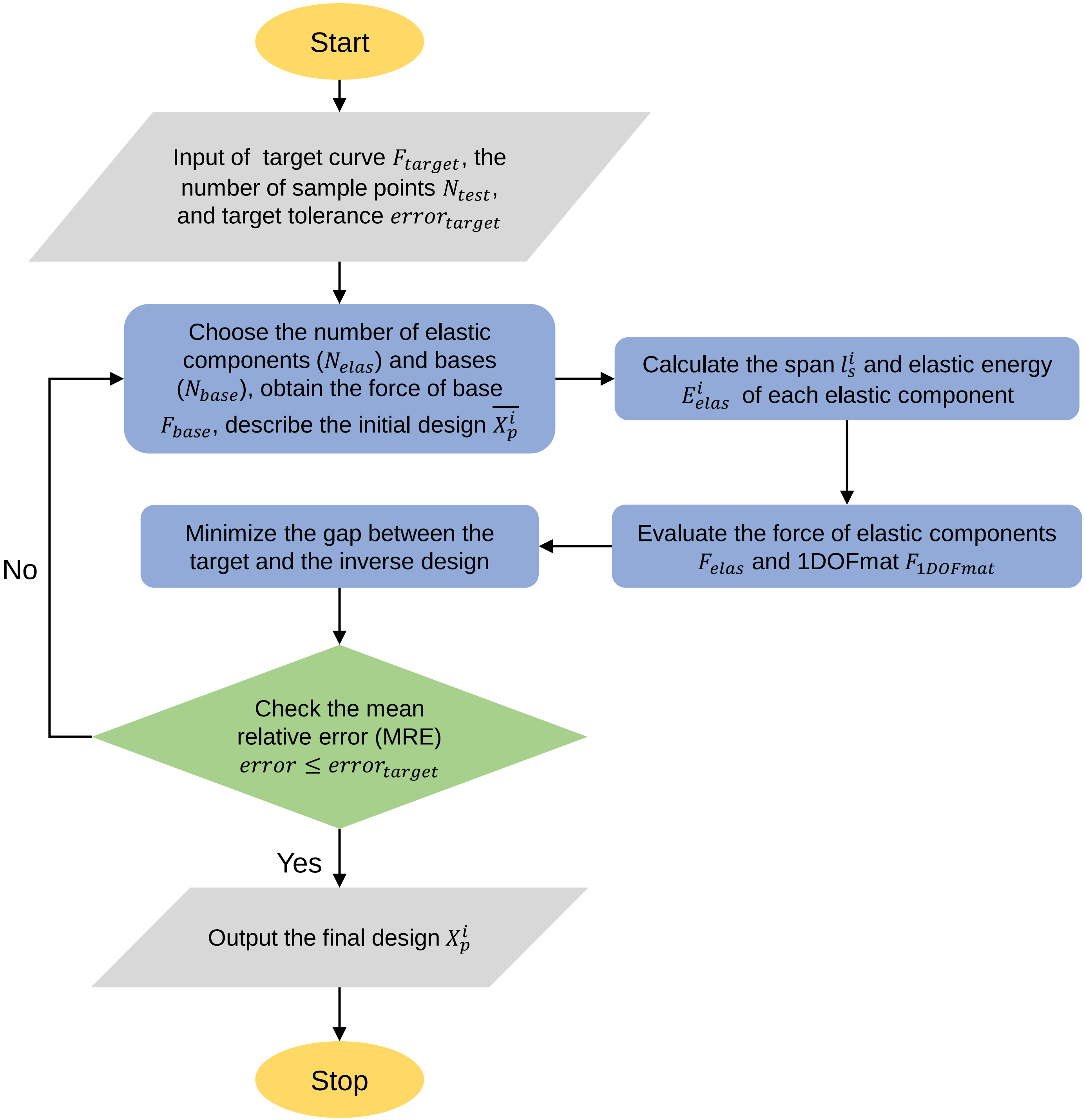

Figure S10. Inverse design process.

decomposing the target curve into N_{test} test points, and minimizing the difference between the 1DOFmat metamaterial force and target force as the optimization objective:

$$\mathcal{L} = \sum_{j=1}^{N_{test}} \left(F_{1DOFmat}^{j} - F_{target}^{j}\right)^2, \tag{S19}$$

using the fmincon function in MATLAB to minimize the sum of squared residuals between the target force and the 1DOFmat theoretical model, the solution with errors smaller than the allowed target error $error_{target}$ is obtained as the final design parameter $X_p^i = [N_1, N_2, u_1, v_1, u_2, v_2, L_0, w]$.

S4.2 A simple example of the inverse design

For a complete understanding of the reverse design process, detailed inverse solution steps are provided for the one-step function curve.

Step 1: Input of target curve F_{target}

First, we define the target curve for the inverse solution. The target force curve is a step function (see Fig. 4*A* in the main manuscript and Movie S3 in the Supporting Information):

$$F_{target} = \begin{cases} 1.436\, d_{disp}, & if \quad 0 \le d_{disp} < 9.75, \\ 14, & if \quad 9.75 \le d_{disp} \le 64.5, \\ 0.333\,(d_{disp} - 64.5), & if \quad 64.5 < led_{disp} \le 75, \end{cases} \tag{S20}$$

selecting 10 sample points for fitting while avoiding the initial segment of the curve due to zero drift, and defining the allowable target error indicator:

$$d_{disp}^{j} = [7.5,\ 14.6,\ 21.7,\ 28.7,\ 35.8,\ 42.9,\ 50.0, 57.1,\ 64.2,\ 71.2], \tag{S21}$$

$$F_{target}^{j} = [10.8,\ 14.0,\ 14.0,\ 14.0,\ 14.0,\ 14.0,\ 14.0,\ 14.0,\ 14.0,\ 16.2], \tag{S22}$$

$$error_{target} = 6\,\%. \tag{S23}$$

Step 2: Calculate the force of elastic components F_{elas}

The initial selection involves one elastic component ($N_{elas} = 1$) and two 1-DOF kinematic bases ($N_{base} = 2$), the reaction force curve F_{base} is obtained through experimental testing. The random topology connections of the elastic component are N_1=31 and N_2=10, the local coordinates for the topology with indices 31 and 10 are transformed into the global coordinate system:

$$\begin{aligned} x_1 &= (b - v_1)\sin\alpha^j + u_1\cos\alpha^j \\ y_1 &= (u_1 + 4a)\sin\alpha^j + (v_1 + 5b)\cos\alpha^j, \\ x_2 &= (v_2 + 3b)\sin\alpha^j + (u_2 + 3a)\cos\alpha^j, \\ y_2 &= (a - u_2)\sin\alpha^j + (v_2 + b)\cos\alpha^j, \end{aligned} \tag{S24}$$

calculating the distance l_s and compression deformation Δl of the spring steel in elastic component:

$$l_s = \sqrt{(x_1 - x_2)^2 + (y_1 - y_2)^2}, \tag{S25}$$

$$\Delta l = L_0 - l_s \tag{S26}$$

where L_0 represents the rest length of the spring steel, and the buckling force P_{elas} for each spring steel element is determined using the rod buckling theory:

$$P_{elas} = P_{cr}\left(1 + \frac{\Delta l}{2L_0}\right), \tag{S27}$$

where P_{cr} is the critical buckling force of the free-end rod $P_{cr} = \frac{\pi^2 EI}{L_0^2}$, E is the Young's modulus of the spring steel, $I = \frac{wt^3}{12}$ is the material's moment of inertia, w is the width of the spring steel, t is the thickness of the spring steel. Metamaterial compression bends the spring steel, generating elastic energy:

$$E_{elas} = P_{cr}\,\Delta l + \frac{P_{cr}\,\Delta l^2}{4L_0}, \tag{S28}$$

the force F_{base} added to the elastic potential energy change E_{elas}, when projected onto the displacement load d_{disp}, is equal to the metamaterial force $F_{1DOFmat}$.

$$F_{1DOFmat} = F_{base} + \frac{\mathrm{d}E_{elas}}{\mathrm{d}d_{disp}}. \tag{S29}$$

Step3: Minimize the gap between the target and the inverse design.
Decompose the target curve into 10 target points and establish the minimization of the difference between metamaterial force and target force as the optimization objective:

$$\mathcal{L} = \sum_{j=1}^{10} \left(F^j_{1DOFmat} - F^j_{target})\right)^2, \tag{S30}$$

utilize the fmincon function in MATLAB to find the optimal fitting parameters by minimizing the sum of squared residuals between the target force and the model predictions, the theoretical force and error associated with the obtained inverse design $X^i_p = (N_1, N_2, u_1, v_1, u_2, v_2, L_0, w)$ are as follows:

$$X^i_p = [32,\ 10,\ 44.283,\ -45.51,\ 25.143,\ 8.804,\ 96.056,\ 6.381], \tag{S31}$$

$$F^j_{1DOFmat} = [10.5,\ 13.9,\ 14.2,\ 14.2,\ 14.1,\ 14.0,\ 14.3,\ 14.4,\ 14.9,\ 16.4], \tag{S32}$$

$$error_{1DOFmat} = 2\,\%, \tag{S33}$$

the error $error_{1DOFmat}$ of this inverse solution is less than the allowable error $error_{target}$, satisfying the design criteria.

Step4: Manufacturing and Experimental Validation
Using the design variables X_p calculated from the inverse solution, create the 3D configuration of the elastic component using SolidWorks. Then, use the Ultimaker S5 for 3D printing, test the force-displacement curve of the designed metamaterial, and extract experimental forces at 10 sample points:

$$F^j_{exp} = [10.0,\ 13.7,\ 14.7,\ 15.1,\ 15.3,\ 15.0,\ 14.2,\ 13.5,\ 13.3,\ 15.3], \tag{S34}$$

$$error_{exp} = 5.3\,\%, \tag{S35}$$

the experimental testing error $error_{exp}$ is very close to the theoretical model error $error_{1DOFmat}$, both of which are smaller than the target allowable error $error_{target}$, this validates the effectiveness of the 1DOFmat model.

S4.3 Inverse design of anisotropic responses

The inverse design of anisotropic responses follows a procedure similar to that in Section S4.2, the key difference being the inclusion of two target curves. The distance l_s and the compression deformation Δl of the spring steel are calculated in both directions X and Y, applying the least squares method minimizes the difference between the target and theoretical responses, ensuring optimal fitting in both directions. Three sets of inverse solution responses are shown in Figure S11 (see Code S4, Code S5 and Code S6 in the Supporting Information).

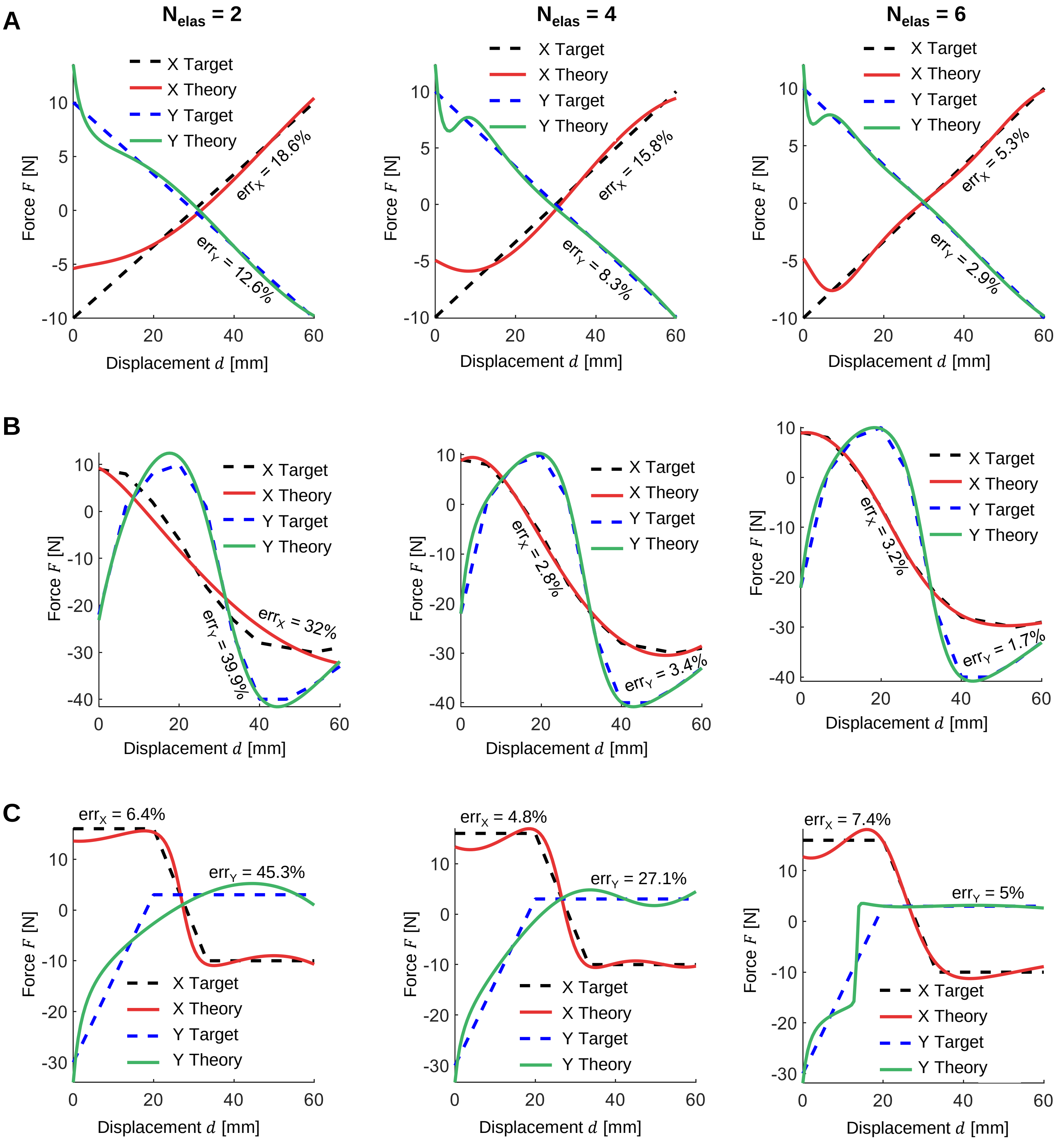

Figure S11. Three sets of inverse design curves for anisotropic responses with different numbers of elastic components. (**A**) Positive linear curve in the X-direction and negative linear curve in the Y-direction. (**B**) Negative linear curve in the X-direction and sinusoidal curve in the Y-direction. (**C**) Two-step descending curve in the X-direction and zero stiffness curve in the Y-direction.

S5. Formulas block

Formulas for the transformation from local coordinates to global coordinates are as follows:

$$N = 01,\quad x = v\sin\alpha + u\cos\alpha,\quad y = -u\sin\alpha + v\cos\alpha;$$
$$N = 02,\quad x = (2b - v)\sin\alpha + (u + a)\cos\alpha,\quad y = (u - a)\sin\alpha + v\cos\alpha;$$
$$N = 03,\quad x = (v + 2b)\sin\alpha + (u + 2a)\cos\alpha,\quad y = -u\sin\alpha + v\cos\alpha;$$
$$N = 04,\quad x = (4b - v)\sin\alpha + (u + 3a)\cos\alpha,\quad y = (u - a)\sin\alpha + v\cos\alpha;$$
$$N = 05,\quad x = (v + 4b)\sin\alpha + (u + 4a)\cos\alpha,\quad y = -u\sin\alpha + v\cos\alpha;$$
$$N = 06,\quad x = (6b - v)\sin\alpha + (u + 5a)\cos\alpha,\quad y = (u - a)\sin\alpha + v\cos\alpha;$$
$$N = 07,\quad x = (b - v)\sin\alpha + u\cos\alpha,\quad y = u\sin\alpha + (v + b)\cos\alpha;$$
$$N = 08,\quad x = (v + b)\sin\alpha + (u + a)\cos\alpha,\quad y = (a - u)\sin\alpha + (v + b)\cos\alpha;$$
$$N = 09,\quad x = (3b - v)\sin\alpha + (u + 2a)\cos\alpha,\quad y = u\sin\alpha + (v + b)\cos\alpha;$$
$$N = 10,\quad x = (v + 3b)\sin\alpha + (u + 3a)\cos\alpha,\quad y = (a - u)\sin\alpha + (v + b)\cos\alpha;$$
$$N = 11,\quad x = (5b - v)\sin\alpha + (u + 4a)\cos\alpha,\quad y = u\sin\alpha + (v + b)\cos\alpha;$$
$$N = 12,\quad x = (v + 5b)\sin\alpha + (u + 5a)\cos\alpha,\quad y = (a - u)\sin\alpha + (v + b)\cos\alpha;$$
$$N = 13,\quad x = v\sin\alpha + u\cos\alpha,\quad y = (2a - u)\sin\alpha + (v + 2b)\cos\alpha;$$
$$N = 14,\quad x = (2b - v)\sin\alpha + (u + a)\cos\alpha,\quad y = (u + a)\sin\alpha + (v + 2b)\cos\alpha;$$
$$N = 15,\quad x = (v + 2b)\sin\alpha + (u + 2a)\cos\alpha,\quad y = (2a - u)\sin\alpha + (v + 2b)\cos\alpha;$$
$$N = 16,\quad x = (4b - v)\sin\alpha + (u + 3a)\cos\alpha,\quad y = (u + a)\sin\alpha + (v + 2b)\cos\alpha;$$
$$N = 17,\quad x = (v + 4b)\sin\alpha + (u + 4a)\cos\alpha,\quad y = (2a - u)\sin\alpha + (v + 2b)\cos\alpha;$$
$$N = 18,\quad x = (6b - v)\sin\alpha - (u + 5a)\cos\alpha,\quad y = (u + a)\sin\alpha + (v + 2b)\cos\alpha;$$
$$N = 19,\quad x = (b - v)\sin\alpha + u\cos\alpha,\quad y = (u + 2a)\sin\alpha + (v + 3b)\cos\alpha;$$
$$N = 20,\quad x = (v + b)\sin\alpha + (u + a)\cos\alpha,\quad y = (3a - u)\sin\alpha + (v + 3b)\cos\alpha;$$
$$N = 21,\quad x = (-v + 3b)\sin\alpha + (u + 2a)\cos\alpha,\quad y = (u + 2a)\sin\alpha + (v + 3b)\cos\alpha;$$
$$N = 22,\quad x = (v + 3b)\sin\alpha + (u + 3a)\cos\alpha,\quad y = (3a - u)\sin\alpha + (v + 3b)\cos\alpha;$$
$$N = 23,\quad x = (5b - v)\sin\alpha + (u + 4a)\cos\alpha,\quad y = (u + 2a)\sin\alpha + (v + 3b)\cos\alpha;$$
$$N = 24,\quad x = (v + 5b)\sin\alpha + (u + 5a)\cos\alpha,\quad y = (3a - u)\sin\alpha + (v + 3b)\cos\alpha;$$
$$N = 25,\quad x = v\sin\alpha + u\cos\alpha,\quad y = (4a - u)\sin\alpha + (v + 4b)\cos\alpha;$$
$$N = 26,\quad x = (2b - v)\sin\alpha + (u + a)\cos\alpha,\quad y = (u + 3a)\sin\alpha + (v + 4b)\cos\alpha;$$
$$N = 27,\quad x = (v + 2b)\sin\alpha + (u + 2a)\cos\alpha,\quad y = (4a - u)\sin\alpha + (v + 4b)\cos\alpha;$$
$$N = 28,\quad x = (4b - v)\sin\alpha + (u + 3a)\cos\alpha,\quad y = (u + 3a)\sin\alpha + (v + 4b)\cos\alpha;$$
$$N = 29,\quad x = (v + 4b)\sin\alpha + (u + 4a)\cos\alpha,\quad y = (4a - u)\sin\alpha + (v + 4b)\cos\alpha;$$
$$N = 30,\quad x = (6b - v)\sin\alpha + (u + 5a)\cos\alpha,\quad y = (u + 3a)\sin\alpha + (v + 4b)\cos\alpha;$$
$$N = 31,\quad x = (b - v)\sin\alpha + u\cos\alpha,\quad y = (u + 4a)\sin\alpha + (v + 5b)\cos\alpha;$$
$$N = 32,\quad x = (v + b)\sin\alpha + (u + a)\cos\alpha,\quad y = (5a - u)\sin\alpha + (v + 5b)\cos\alpha;$$
$$N = 33,\quad x = (3b - v)\sin\alpha + (u + 2a)\cos\alpha,\quad y = (u + 4a)\sin\alpha + (v + 5b)\cos\alpha;$$
$$N = 34,\quad x = (v + 3b)\sin\alpha + (u + 3a)\cos\alpha,\quad y = (5a - u)\sin\alpha + (v + 5b)\cos\alpha;$$
$$N = 35,\quad x = (5b - v)\sin\alpha + (u + 4a)\cos\alpha,\quad y = (u + 4a)\sin\alpha + (v + 5b)\cos\alpha;$$
$$N = 36,\quad x = (v + 5b)\sin\alpha + (u + 5a)\cos\alpha,\quad y = (5a - u)\sin\alpha + (v + 5b)\cos\alpha;$$

References

[1] M. Hägele, K. Nilsson, J. N. Pires, R. Bischoff, *Springer handbook of robotics* **2016**, 1385–1422.

[2] D. P. Bertsekas, *Constrained optimization and Lagrange multiplier methods*, Academic press, **2014**.

[3] L. Cedolin, et al., *Stability of structures: elastic, inelastic, fracture and damage theories*, World Scientific, **2010**.
